# Antibacterial activity of enmetazobactam against *Acinetobacter* spp.: a molecular dissection of mechanism of action and resistance determinants

**DOI:** 10.1128/aac.01206-25

**Published:** 2025-12-22

**Authors:** Gabriela-Alejandra Báez-Barroso, Arianna Rodríguez-Coello, Juan Carlos Vázquez-Ucha, Silvia López-Argüello, Michelle Outeda-García, Lucía González-Pinto, Andrea García-Pose, Paula Guijarro-Sánchez, Isaac Alonso-García, Emilio Lence, Concepción González-Bello, Antonio Oliver, Jorge Arca-Suárez, Bartolome Moya, Germán Bou, Alejandro Beceiro, on behalf of the Spanish National Acinetobacter spp. 2020 Study Group

**Affiliations:** 1 Servicio de Microbiología and Instituto de Investigación Biomédica A Coruña (INIBIC), Complexo Hospitalario Universitario A Coruña (CHUAC) 16811, A Coruña, Spain; 2 Ciber de Enfermedades Infecciosas (CIBERINFEC), Instituto de Salud Carlos III 38176, Madrid, Spain; 3 Servicio de Microbiología and Unidad de Investigación, Hospital Universitario Son Espases, Health Research Institute of the Balearic Islands (IdISBa) 375118, Palma, Spain; 4 Centro Singular de Investigación en Química Biolóxica e Materiais Moleculares (CiQUS), Departamento de Química Orgánica, Universidade de Santiago de Compostela 16780, Santiago de Compostela, Spain; 5 Departamento de Química Orgánica, Facultad de Ciencias, Universidad de Valladolid 201461 , Valladolid, Spain; 6 Departamento de Fisioterapia, Medicina y Ciencias Biomédicas, Universidad de A Coruña 16737 , A Coruña, Spain; University of Fribourg, Fribourg, Switzerland

**Keywords:** antimicrobial resistance, cefepime/enmetazobactam, sulbactam, durlobactam, *Acinetobacter *spp., *Acinetobacter baumannii*, PBPs, carbapenem-hydrolyzing class D β-lactamases

## Abstract

The persistence of multidrug-resistant *Acinetobacter baumannii* remains a clinical challenge. Cefepime/enmetazobactam is a novel combination with demonstrated activity against extended-spectrum β-lactamase-producing Enterobacterales, but its activity against *Acinetobacter* has not yet been thoroughly explored. We aimed to assess its activity against *Acinetobacter* spp., including multidrug-resistant strains producing carbapenem-hydrolyzing class D β-lactamases (CHDLs). We analyzed 208 clinical isolates of *Acinetobacter* spp., including 67 carbapenem-resistant *Acinetobacter baumannii* (CRAB). Antibiotic susceptibility testing was conducted with cefepime, sulbactam, and imipenem, alone and in combination with enmetazobactam; the latter was also tested individually. Additionally, MICs of enmetazobactam/durlobactam and sulbactam/durlobactam were determined for CRAB and CHDL-producing *A. baumannii* ATCC 17978 transformants. PBP binding assays (IC₅₀), molecular docking, simulation studies with the enmetazobactam/OXA-23 adduct, hydrolysis kinetics (*k*_cat_, *K*_m_), and OXA-23 inhibition assays (IC₅₀, *k*_off_, *t*_₁/₂_) were performed to elucidate the mechanism of enmetazobactam and detect reduced susceptibility. Enmetazobactam showed high intrinsic activity against *Acinetobacter* spp., displaying reduced MICs against carbapenem-susceptible isolates. MIC_50/90_ of the enmetazobactam/durlobactam combination was 2/2 mg/L for CHDL-producing *A. baumannii*. Enmetazobactam exhibited bactericidal activity comparable to sulbactam. Binding assays revealed that the antimicrobial activity is driven by selective affinity for PBP2 (IC₅₀ 3.6 mg/L) and PBP3 (IC₅₀ 4.2 mg/L). OXA-23 readily inactivated enmetazobactam, confirming the major role of CHDLs in resistance to enmetazobactam, via substrate-assisted de-acylation. This study evidences the potent antimicrobial activity of enmetazobactam against *A. baumannii* via inhibition of PBP2 and PBP3. Its combination with new OXA-type inhibitors (e.g., durlobactam) represents a potential therapeutic alternative for multidrug-resistant *A. baumannii*.

## INTRODUCTION

The genus *Acinetobacter* includes several species of opportunistic gram-negative bacteria that have been identified as important pathogens in hospital settings. *Acinetobacter baumannii* stands out among these bacteria for its particular clinical relevance, due to its capacity to cause severe infections, especially in critically ill patients, and its ability to survive in hospital environments and its intrinsic resistance to disinfectants (1). This microorganism is responsible for infections such as ventilator-associated pneumonia, bacteremia, wound infections and urinary tract infections.

The high capacity of *A. baumannii* to develop resistance to multiple antimicrobial families is a cause for great concern. Treatment options are currently severely limited and frequently ineffective, mainly due to acquired resistance mechanisms mediated by carbapenem-hydrolyzing class D β-lactamases (CHDLs). Traditional therapeutic options include carbapenems, the efficacy of which has been compromised by CHDLs (2, 3), and colistin, used as a last-line defense but associated with adverse, toxic effects (4, 5). New antimicrobial agents, such as cefiderocol and the sulbactam/durlobactam combination, have shown promising activity against multidrug-resistant *A. baumannii* strains and represent significant advances in therapy (1, 6). However, resistance to these treatments has already been described (7–9). Thus, in light of the limited number of therapeutic options, *A. baumannii* has been categorized as a critical priority in the WHO Bacterial Priority Pathogens List (10).

**Fig 1:**
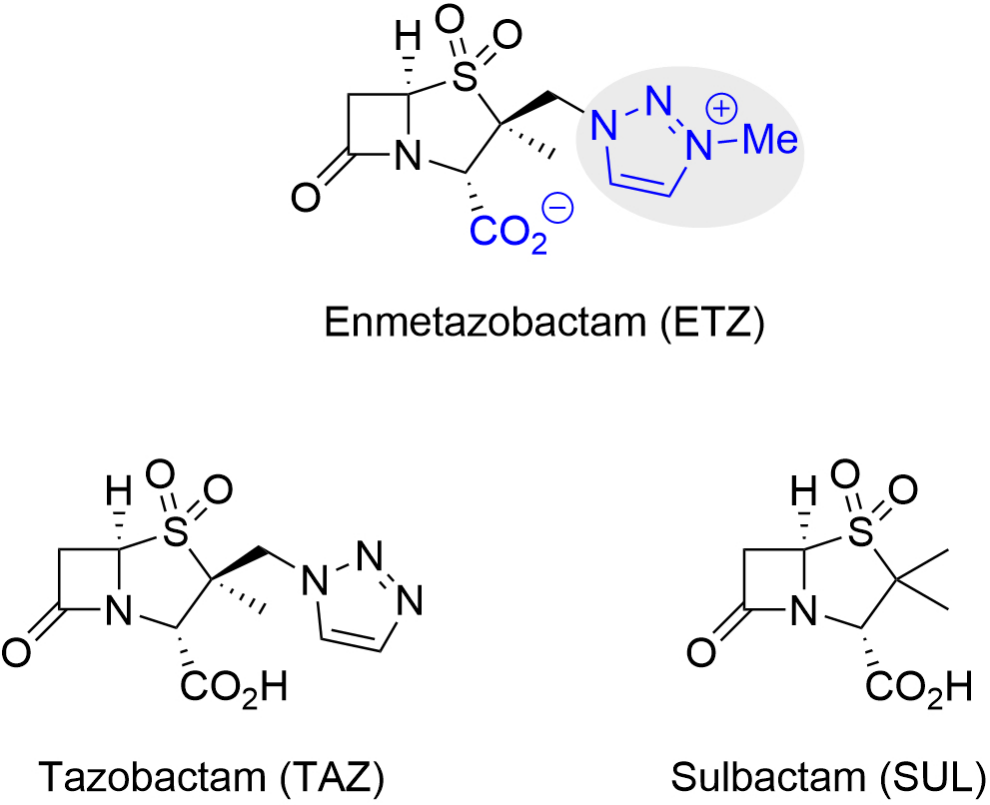
Chemical structures of enmetazobactam (ETZ) and its parent compounds tazobactam (TAZ) and sulbactam (SUL).

Among the newly available therapeutic options for multidrug-resistant pathogens, the cefepime/enmetazobactam combination has emerged as a promising treatment for infections caused by extended-spectrum β-lactamase-producing Enterobacterales (11). Approved by the FDA and EMA in 2024, this formulation pairs a fourth-generation cephalosporin with enmetazobactam, a novel β-lactamase inhibitor resulting from methylation of the terminal nitrogen atom in tazobactam. This modification confers enhanced bacterial cell penetration and an expanded spectrum of inhibition against cephalosporinases, which are attributed to its zwitterionic character, also present in its drug partner (Fig. 1) (12). The development of cefepime/enmetazobactam has primarily focused on Enterobacterales; thus, its intrinsic antimicrobial and inhibitory activity against β-lactamases of *Acinetobacter* spp. remains to be elucidated. Although, a priori, a low efficacy might be expected due to known resistance mechanisms in this genus, we hypothesized that enmetazobactam could exert additional antibacterial effects, beyond β-lactamase inhibition, such as penicillin-binding protein (PBP) targeting or synergistic enhancement of β-lactam activity, as previously described for other β-lactamase inhibitors (e.g., durlobactam or zidebactam) with dual mechanisms of action (13, 14).

The overall objective of this study was to evaluate the antimicrobial activity of cefepime/enmetazobactam combination and enmetazobactam alone against *Acinetobacter* spp. The study also aimed to identify the mode of action of enmetazobactam and any resistance mechanism that would limit its applicability. The activity of this compound was assessed to support improved treatment strategies against this challenging pathogen.

## RESULTS AND DISCUSSION

### Enmetazobactam reveals bactericidal activity against *Acinetobacter* spp. in the absence of carbapenemases

The 208 isolates from the 2020 multicenter study of *Acinetobacter* spp. were classified into three groups for analysis: (i) carbapenem-resistant strains, with the presence of acquired OXA-type carbapenemases in most cases (*n* = 67), (ii) carbapenem-susceptible strains of *A. baumannii* (*n* = 54), and (iii) *Acinetobacter* non-*baumannii* strains (*n* = 87). The MICs of cefepime, imipenem, and sulbactam alone and in combination with enmetazobactam (fixed concentration of 8 mg/L), as well as of enmetazobactam alone, were determined. Detailed susceptibility values for each individual isolate are provided in Tables S1 to S3.

The carbapenem-resistant *A. baumannii* (CRAB) isolates yielded high MICs for imipenem, cefepime, and sulbactam (Table 1). The addition of enmetazobactam modestly improved the activity of all three antibiotics, decreasing the MIC_50/90_ values by 0–1 dilutions. Sulbactam/enmetazobactam was the most active combination in this context, with a MIC_50/90_ of 8/16 mg/L. However, these values indicate the limited activity of these therapeutic combinations against carbapenem-resistant strains. The MIC_50/90_ for enmetazobactam alone was 64/64 mg/L. Thus, the data revealed poor potentiation of sulbactam, cefepime, and imipenem by enmetazobactam and also the low antibacterial activity of enmetazobactam alone against CRAB. Of note, these results are consistent with those reported by Liu *et al*. (15), who assessed the antimicrobial properties of cefepime/enmetazobactam in 255 clinical isolates of carbapenem-non-susceptible *A. baumannii* and reported MIC_50/90_ values of 128/>128 mg/L for cefepime and >64/>64 mg/L for the cefepime/enmetazobactam combination. Similar results were obtained for cefepime/enmetazobactam when tested against a collection of CRAB isolates by Bonnin *et al*. (16).

**TABLE 1:** MIC (mg/L) distributions and resistance rates to the tested antibiotics relative to the CRAB isolates (*n* = 67)^*a*^

β-lactams	MIC distribution												MIC_50_	MIC_90_
	≤0.06	0.12	0.25	0.5	1	2	4	8	16	32	64	≥128		
FEP	0	0	0	0	0	0	1.5	3.0	22.4	70.1	97.0	100	32	64
FEP/ETZ (8 mg/L)	14.9	14.9	14.9	14.9	14.9	16.4	17.9	28.4	46.3	95.5	100	100	32	32
IMI	0	0	0	0	0	0	0	9.0	34.3	79.1	100	100	32	64
IMI/ETZ (8 mg/L)	14.9	14.9	14.9	14.9	14.9	16.4	17.9	26.9	59.7	91.0	100	100	16	32
SUL	0	0	0	0	0	14.9	17.9	34.3	89.6	100	100	100	16	32
SUL/ETZ (8 mg/L)	14.9	14.9	14.9	14.9	16.4	17.9	28.4	61.2	98.5	100	100	100	8	16
ETZ	0	0	0	0	0	0	10.4	19.4	25.4	41.8	100	100	64	64

^*a*^ FEP, cefepime; ETZ, enmetazobactam; IMI, imipenem; SUL, sulbactam.

In the group of carbapenem-susceptible *A. baumannii* isolates, the β-lactam/enmetazobactam combinations exhibited excellent antimicrobial activity (Table 2). In initial testing, MIC_50/90_ values of ≤0.06/≤0.06 mg/L were obtained for cefepime/enmetazobactam, imipenem/enmetazobactam, and sulbactam/enmetazobactam, indicating complete inhibition of growth. The individual values for cefepime, imipenem, and sulbactam were also favorable (MIC_90_ of 8, 0.25, and 2 mg/L, respectively), although they were clearly enhanced by the addition of enmetazobactam. Enmetazobactam alone demonstrated strong antimicrobial activity (MIC_50/90_ of 2/4 mg/L), very similar to that of sulbactam (MIC_50/90_ of 1/2 mg/L). This observation underlies the complete growth inhibition observed in the MIC assays of antibiotics combined with enmetazobactam. These findings identify enmetazobactam as a β-lactamase inhibitor with intrinsic antibacterial activity against *A. baumannii*, revealing a previously unrecognized and overlooked therapeutic potential.

**TABLE 2:** MIC (mg/L) distributions and resistance rates to the tested antibiotics relative to the carbapenem-susceptible *A. baumannii* isolates (*n* = 54)^*b*^

β-lactams	MIC distribution										MIC_50_	MIC_90_
	≤0.06	0.12	0.25	0.5	1	2	4	8	16	≥32		
FEP	0	0	0	0	13.0	44.4	87.0	94.4	98.1	100	4	8
FEP/ETZ (8 mg/L)	100	100	100	100	100	100	100	100	100	100	≤0.06^*a*^	≤0.06^*a*^
IMI	11.1	61.1	98.1	98.1	100	100	100	100	100	100	0.12	0.25
IMI/ETZ (8 mg/L)	100	100	100	100	100	100	100	100	100	100	≤0.06^*a*^	≤0.06^*a*^
SUL	0	0	0	27.8	79.6	96.3	100	100	100	100	1	2
SUL/ETZ (8 mg/L)	100	100	100	100	100	100	100	100	100	100	≤0.06^*a*^	≤0.06^*a*^
ETZ	0	0	0	0	3.7	51.9	96.3	100	100	100	2	4

^*a*^ Complete growth inhibition due to the enmetazobactam concentration (8 mg/L) used when combined with antibiotics.

^*b*^ FEP, cefepime; ETZ, enmetazobactam; IMI, imipenem; SUL, sulbactam.

Finally, in the *A*. non-*baumannii* group, the activity of β-lactams in combination with enmetazobactam was again excellent, with a MIC_50/90_ of ≤0.06/≤0.06 mg/L for cefepime/enmetazobactam, imipenem/enmetazobactam, and sulbactam/enmetazobactam combinations (Table 3). Although cefepime, imipenem, and sulbactam alone presented reasonable antibacterial efficacy (MIC_90_ of 4, 0.25, and 2 mg/L, respectively), the addition of enmetazobactam substantially enhanced the potency of these compounds, with complete inhibition of all isolates. Enmetazobactam alone again demonstrated clear antimicrobial activity (MIC_50/90_ of 2/4 mg/L).

**TABLE 3:** MIC (mg/L) distributions and resistance rates to the tested antibiotics relative to the *A*. non*-baumannii* isolates (*n*=87)^*b*^

β-lactams	MIC distribution										MIC_50_	MIC_90_
	≤0.06	0.12	0.25	0.5	1	2	4	8	16	32		
FEP	0	1.1	4.6	12.6	41.4	73.6	96.6	98.9	100	100	2	4
FEP/ETZ (8 mg/L)	98.9	98.9	98.9	98.9	98.9	100	100	100	100	100	≤0.06^*a*^	≤0.06^*a*^
IMI	32.2	71.3	96.6	97.7	97.7	97.7	97.7	97.7	97.7	100	0.12	0.25
IMI/ETZ (8 mg/L)	98.9	98.9	98.9	98.9	98.9	98.9	98.9	100	100	100	≤0.06^*a*^	≤0.06^*a*^
SUL	1.1	2.3	8.0	44.8	82.8	97.7	100	100	100	100	1	2
SUL/ETZ (8 mg/L)	98.9	98.9	98.9	98.9	100	100	100	100	100	100	≤0.06^*a*^	≤0.06^*a*^
ETZ	0	0	1.1	9.2	29.9	75.9	96.6	98.9	100	100	2	4

^*a*^ Complete growth inhibition due to the enmetazobactam concentration (8 mg/L) used when combined with antibiotics.

^*b*^ FEP, cefepime; ETZ, enmetazobactam; IMI, imipenem; SUL, sulbactam.

These highly favorable susceptibility profiles in *A. non-baumannii* are consistent with those observed in *A. baumannii* carbapenem-susceptible isolates, demonstrating the robust efficacy of enmetazobactam combinations against strains that do not produce CHDLS, such as OXA-23, OXA-24/40, OXA-58, or over-expressed OXA-51-like carbapenemases. Importantly, this represents a novel and previously unreported finding, as the activity of enmetazobactam in *Acinetobacter* species, particularly non-*baumannii* isolates, has not been systematically studied before. These results hold significant clinical relevance given that advances in diagnostics have led to increased identification of some non-*baumannii* species, such as *A. pittii*, which in some settings surpasses *A. baumannii* in blood cultures (17), and increasing reports of multidrug-resistant strains highlight the growing clinical significance of this species (18).

Importantly, the improved potency observed with the combinations including enmetazobactam does not appear to be attributed to inhibition of β-lactamases but rather to the unexpected intrinsic antimicrobial activity of enmetazobactam against the genus *Acinetobacter*. In these combinations, enmetazobactam was used at a fixed concentration of 8 mg/L, which is equal to or higher than the MICs determined in these assays for all strains in the *A. baumannii* carbapenem-susceptible and *A*. non-*baumannii* groups (Tables S2 and S3). This observation suggests that the apparent enhancement of the activity of β-lactam antibiotics (cefepime, imipenem, and sulbactam) actually reflects direct susceptibility to enmetazobactam, although the effectiveness seems to be limited by the presence of OXA-like carbapenemases such as OXA-23, OXA-24/40, or OXA-58 (Table S1).

The minimum bactericidal concentrations (MBCs) of enmetazobactam and sulbactam (as comparator) for nine clinical isolates were also determined (Table S4). Overall, the MBC was very similar to the MIC for both compounds, being the same or up to twofold higher for most of the isolates (only a non-CHDLs-producing *A. baumannii* isolate yielded an enmetazobactam MBC eightfold higher than the MIC). The close correspondence between MBC and MIC values suggests that enmetazobactam not only inhibits bacterial growth but also exerts potent bactericidal effects at comparable concentrations, closely aligned with sulbactam. To our knowledge, this is the first study to evaluate and report the bactericidal properties of enmetazobactam against *Acinetobacter* spp., representing a novel and clinically meaningful contribution to the understanding of its antimicrobial potential.

### Lack of synergistic activity between enmetazobactam and established therapies against *A. baumannii*

Checkerboard assays were conducted to evaluate potential synergistic interactions between enmetazobactam and either sulbactam or colistin. MICs of single compounds and combinations were determined, and fractional inhibitory concentration (FIC) indices were calculated for three carbapenem-resistant CHDL-producing *A. baumannii* isolates (OXA-23, OXA-24/40, and OXA-58). However, no synergistic interactions were observed, with the FIC_index_ values consistently being >0.5 (Table S5). Similarly, combining cefepime/enmetazobactam (8 mg/L) with sulbactam also yielded FIC indices >0.5 across all isolates. Therefore, no synergy between the mechanism of action of enmetazobactam and that of sulbactam (inhibition of PBPs) or colistin (cell membrane disruption) was found.

### PBP2 and PBP3 are the molecular targets of enmetazobactam in *A. baumannii*

**Fig 2:**
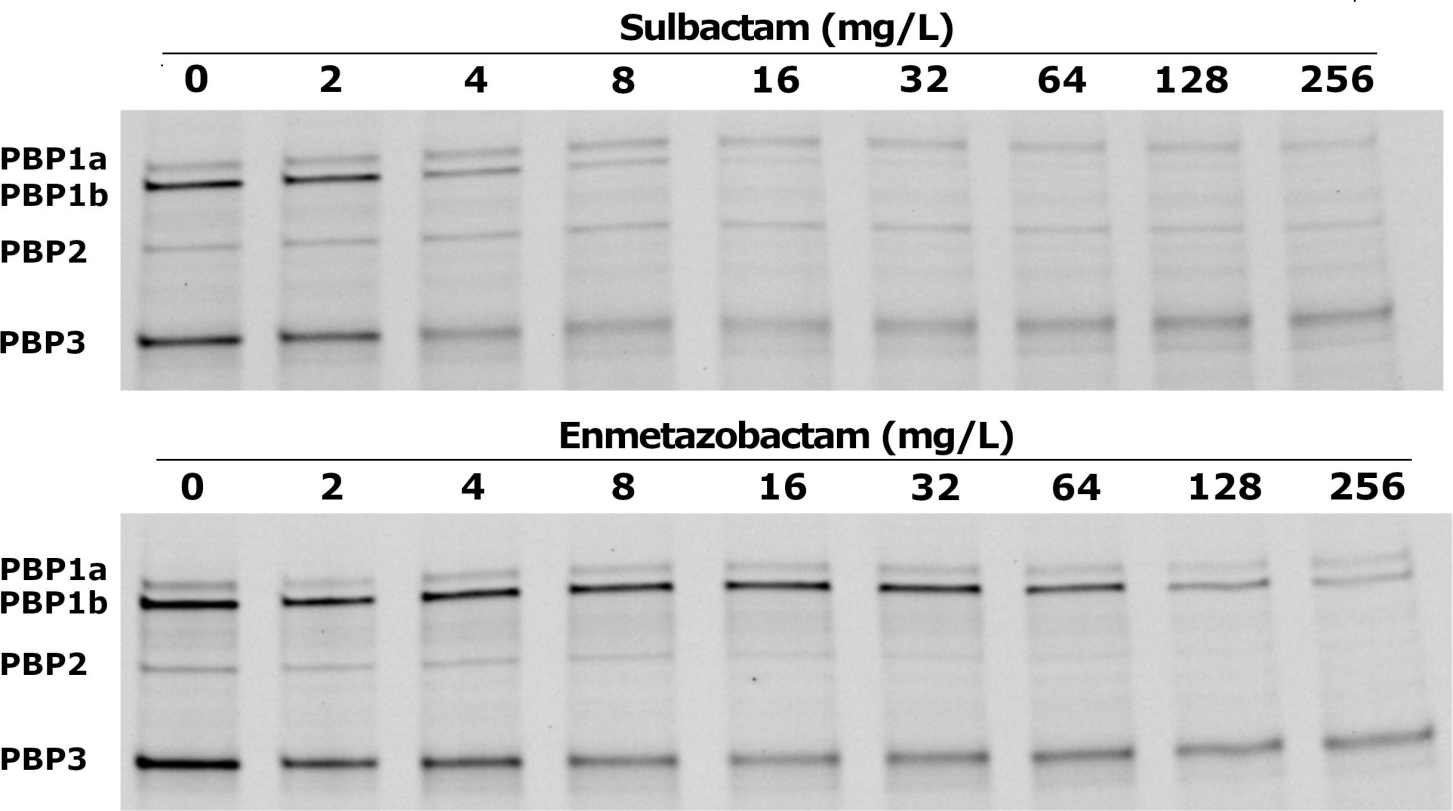
Representative assay of a PBP-binding IC_50_ SDS-polyacrylamide gel with increasing amounts of sulbactam and enmetazobactam in competition with Bocillin FL. The identified PBPs of the *A. baumannii* strain ATCC 19606 are indicated in the figure.

To better understand the mechanism and targets of enmetazobactam against *Acinetobacter* spp., two different assays measuring the possible interaction with PBPs were performed. On the one hand, PBP binding of membrane fractions obtained from *A. baumannii* strain ATCC 19606 was assessed, using increasing concentrations of enmetazobactam and sulbactam (as methodological control and comparator). The fractions were subsequently labeled with Bocillin FL to determine the β-lactam concentrations that half-maximally inhibited (IC_50_) the Bocillin binding (Fig. 2 and Table 4). Sulbactam displayed high affinity for PBP1b and PBP3, with IC_50_ values of 2.24 ± 0.86 and 2.27 ± 0.82 mg/L, respectively, as previously described (13, 19). Enmetazobactam displayed a significant binding preference for PBP2 and PBP3, with IC_50_ values of 3.62 ± 1.15 and 4.24 ± 1.5 mg/L, respectively, whereas other PBPs had much higher IC_50_ values (>64 mg/L).

**TABLE 4:** Binding affinities (IC_50_ in mg/L) of enmetazobactam and sulbactam for PBPs in membrane preparations from *A. baumannii* ATCC 19606

PBP	IC_50_ sulbactam	IC_50_ enmetazobactam
PBP1a	>64	>64
PBP1b	2.24 ± 0.86	>64
PBP2	>64	3.62 ± 1.15
PBP3	2.27 ± 0.82	4.24 ± 1.5

PBP binding assays conducted in *A. baumannii* revealed that enmetazobactam exhibits high affinity for PBP2 and PBP3, with IC₅₀ values comparable to those obtained for sulbactam. Thus, the intrinsic antibacterial activity of enmetazobactam against *Acinetobacter* spp. seems to be mediated by direct inhibition of essential PBPs, thereby compromising cell wall synthesis.

The inhibition of PBPs is known to change the cellular morphology of gram-negative bacteria. Thus, a second assay involving microscopic analysis of *A. baumannii* ATCC 19606 exposed overnight to enmetazobactam, as well as to sulbactam and imipenem as comparators, was performed (Fig. S1). Thus, exposure to sulbactam 1× MIC led to production of long filaments, as expected due to the inhibition of PBP3 (20). By contrast, exposure to imipenem at 1× MIC led to production of rounded and spherical cells, caused by the inhibition of PBP2 (21). Exposure of *A. baumannii* ATCC 19606 strain to enmetazobactam at 1× MIC led to the production of elongated filaments, indicating that PBP3 was being inhibited, preventing the cell from forming the septum and dividing. However, when the concentration of enmetazobactam was increased to 2× MIC, the filaments shortened in length, which could probably be due to PBP2 inhibition. The initial observation of cell elongation, and some subsequent shortening and spherification as the concentration increased, was probably due to greater inhibition of PBP3 and subsequent inhibition of PBP2 when the concentration of enmetazobactam increases, as illustrated in Fig. 2.

Microscopy-based morphological analysis in the presence of β-lactams was consistent with the findings of the previously described PBP-binding assays, supporting the conclusion that enmetazobactam effectively targets PBPs in *Acinetobacter* spp. This binding leads to functional disruption of these essential proteins, thereby confirming PBP inhibition as the primary mechanism underlying the antimicrobial activity of enmetazobactam. Enmetazobactam is, to date, the second β-lactamase inhibitor reported to exhibit intrinsic antibacterial activity against *Acinetobacter* spp., after sulbactam. Both compounds share activity against PBP3; however, enmetazobactam also targets PBP2, a binding profile that differs from sulbactam and involves a PBP that is not commonly inhibited in this genus.

### Combination of enmetazobactam with a class D β-lactamase inhibitor shows potent activity against carbapenemase-producing *A. baumannii*

The study findings prompted the need for further studies to characterize the activity of enmetazobactam against the difficult-to-treat CRAB. Therefore, the activity of enmetazobactam in combination with durlobactam, a diazabicyclooctane class D β-lactamase inhibitor, was tested with this group of isolates. Sulbactam in combination with durlobactam was approved in 2023 by the FDA for treatment of pneumonia caused by the *Acinetobacter baumannii-calcoaceticus* complex (22). Moreover, durlobactam was also identified as a PBP inhibitor, particularly of PBP2, indicating that it may contribute to intrinsic antibacterial activity against *A. baumannii*, beyond its established role in inhibiting class A, C, and D β-lactamases (23).

The susceptibility to enmetazobactam/durlobactam was compared with that of sulbactam/durlobactam (Table 5). All 67 strains tested displayed clinically relevant resistance mechanisms such us (i) CHDLs (OXA-23, OXA 24/40, or OXA-58) or (ii) OXA-51-like, preceded by the insertion sequence IS*AbaI*, which has been described as containing strong promoters for the expression of this class of β-lactamases (24) (Table S1). The enmetazobactam/durlobactam combination exhibited markedly stronger potency against multidrug-resistant *A. baumannii* strains, with MIC_50/90_ of 2/2 mg/L, compared to enmetazobactam alone (MIC_50/90_ of 64/64 mg/L). Likewise, the *in vitro* activity of sulbactam (MIC_50/90_ of 16/32 mg/L) was also enhanced in the presence of durlobactam (MIC_50/90_ of 1/1 mg/L), as expected. The MIC values for each individual isolate are presented in Table S6. These results demonstrate that the enmetazobactam/durlobactam combination exhibits potent *in vitro* activity, comparable to that of the clinically approved sulbactam/durlobactam treatment. Notably, both enmetazobactam and durlobactam, combined with cefepime and sulbactam, respectively, have already received regulatory approval for clinical use, having successfully completed the required safety and efficacy evaluations. With the necessary clinical trials, the combination enmetazobactam/durlobactam may represent a promising therapeutic candidate that could be translated into clinical practice. Given the limited treatment options currently available for infections caused by CRAB, the identification of a novel, effective combination comprising two already-approved agents represents a clinically relevant and timely advance. Given this outcome, assessment of preclinical *in vivo* efficacy of enmetazobactam/durlobactam is therefore warranted to determine the pharmacokinetic/pharmacodynamic profile and therapeutic impact against CRAB.

**TABLE 5:** MIC distributions (%) and MIC_50/90_ to enmetazobactam and sulbactam combined with durlobactam for the set of CHDLs-producing *A. baumannii* isolates (*n* = 67)^*a*^

β-lactams	MIC (mg/L)											MIC_50_	MIC_90_
	≤0.06	0.12	0.25	0.5	1	2	4	8	16	32	≥64		
ETZ	0.0	0.0	0.0	0.0	0.0	0.0	10.4	22.4	25.4	46.3	100	64	64
ETZ/DUR (4 mg/L)	1.5	1.5	1.5	3.0	7.5	91.0	100	100	100	100	100	2	2
SUL	0.0	0.0	0.0	0.0	0.0	13.4	20.9	34.3	89.6	100	100	16	32
SUL/DUR (4 mg/L)	6.0	9.0	17.9	38.8	91.0	100	100	100	100	100	100	1	1

^*a*^ ETZ, enmetazobactam; DUR, durlobactam; SUL, sulbactam.

In order to confirm the involvement of CHDLs in the resistance to enmetazobactam, its activity was tested with a collection of ATCC 17978 transformants harboring different OXA-like carbapenemase genes. As shown in Table 6, the ATCC 17978 strain expressing the most prevalent CHDLs increased the imipenem MIC values, effectively modeling the primary resistance mechanism observed in clinical *A. baumannii* strains and confirming the suitability of our model. In these ATCC 17978 transformants, the enmetazobactam MIC values increased from 4- to ≥32-fold, relative to the control ATCC 17978 with the empty plasmid pETRA, decreasing substantially in all CHDLs-producing transformants to 1 mg/L when combined with durlobactam. This phenomenon was also reproduced with the sulbactam/durlobactam combination. Altogether, this series of assays established that the presence of OXA-type carbapenemases constitutes the principal mechanism driving enmetazobactam resistance in *A. baumannii*. Furthermore, the co-administration of class D β-lactamase inhibitors effectively restores enmetazobactam activity, mirroring the effect observed with the sulbactam/durlobactam combination.

**TABLE 6:** MIC values (mg/L) for the collection of ATCC 17978 transformants collection expressing OXA-like carbapenemase genes

*A. baumannii* ATCC 17978 transformants	IMI	ETZ	ETZ/DUR^*a*^	SUL	SUL/DUR	FEP	FEP/ETZ
ATCC 17978 + pETRA^*b*^	0.25	2	1	2	≤0.25	4	≤0.25
ATCC 17978 + OXA-51	1	4	1	1	≤0.25	4	≤0.25
ATCC 17978 + OXA-201	8	4	1	2	≤0.25	4	≤0.25
ATCC 17978 + OXA-23	≥32	16	1	4	≤0.25	16	8
ATCC 17978 + OXA-24/40	≥32	16	1	4	≤0.25	4	4
ATCC 17978 + OXA-58	≥32	8	1	4	≤0.25	4	≤0.25
ATCC 17978 + OXA-143	≥32	≥32	1	8	≤0.25	4	4

^*a*^ Durlobactam was used in combination with enmetazobactam and sulbactam at a fixed concentration of 4 mg/L, and enmetazobactam was used in combination with cefepime at a fixed concentration of 8 mg/L. ETZ, enmetazobactam; DUR, durlobactam; SUL, sulbactam; FEP, cefepime; IMI, imipenem.

^*b*^ ATCC 17978 carrying the empty plasmid pETRA without CHDL.

### Decreased stability of enmetazobactam in the presence of OXA-23 carbapenemase

Enzymatic kinetic studies were performed in order to determine whether OXA-type carbapenemases from *A. baumannii* sequester or hydrolyze enmetazobactam. Specifically, to evaluate the potential hydrolysis of enmetazobactam by class D β-lactamases, the kinetic parameters *k*_cat_ and *K*_m_ for OXA-23-mediated hydrolysis were first determined (Table 7). The catalytic efficiency (*k*_cat_/*K*_m_) was 1.03 × 10³ M⁻¹s⁻¹ for enmetazobactam and 1.80 × 10³ M⁻¹s⁻¹ for sulbactam, consistent with previously published data for sulbactam (25). This outcome indicates that the catalytic efficiency of OXA-23 is similar for both compounds, suggesting that enmetazobactam is labile to hydrolysis by this carbapenemase.

**TABLE 7:** Steady-state kinetic constants for hydrolysis of enmetazobactam and sulbactam by OXA-23

Compound	*K*_m_ (μM)	*k*_cat_ (s^−1^)	*k*_cat_/*K*_m_ (M^−1^s^−1^)
Enmetazobactam	171.85 ± 55.78	0.177 ± 0.056	1.03 × 10^3^
Sulbactam	524.25 ± 150.94	0.946 ± 0.27	1.80 × 10^3^

Although the absolute *k*_cat_/*K*_m_ values were relatively low for both enmetazobactam and sulbactam, it is important to consider that previous studies with CHDLs such as OXA-23 have also reported low catalytic efficiencies for carbapenems such as imipenem and meropenem, yet sufficient to confer resistance in *A. baumannii*, increasing the MICs up to 128-fold (26). This supports the hypothesis that even low catalytic efficiency can have a clinically important impact on β-lactam efficacy in pathogens with low permeability, such as *A. baumannii*. Thus, these findings suggest that the hydrolytic activity of OXA-23 against enmetazobactam is sufficient to obtain the above-described MICs increments (about 16- to 32-fold) in the challenging *A. baumannii* background, characterized by low permeability and baseline efflux activity, which significantly limit the accessibility of β-lactams.

Inhibition assays were performed to evaluate the ability of enmetazobactam, sulbactam, and durlobactam to inhibit OXA-23 carbapenemase. As expected, durlobactam showed greater inhibitory capacity (IC₅₀ =0.17 µM) under the test conditions, in line with previous studies with OXA-24/40, a similar CHDL (IC₅₀ =0.19 µM) (23). In contrast, enmetazobactam and sulbactam exhibited significantly higher IC₅₀ values (32.41 and 29.21 µM, respectively), confirming their limited inhibitory capacity against this enzyme (Table S7). Inhibition‐kinetics assays using dilution-triggered dissociation (*k*_off_) assays with OXA-23 were also performed. Jump dilution assays revealed that β-lactamase activity in the presence of enmetazobactam recovered almost instantaneously. As shown in Fig. S2, the post-dilution nitrocefin hydrolysis rate was similar to that of the uninhibited control, precluding accurate determination of *k*_off_. However, the *k*_off_ value for enmetazobactam was >1,000-fold higher than that of durlobactam (*k*_off_ = 2.8 ± 0.028 × 10^−5^ s^−1^), therefore resulting in a drastically shorter residence time than for durlobactam (*t*_1/2_= 412 ±4 min), which displayed a much more prolonged interaction, consistent with its efficacy as a class D carbapenemase inhibitor (27).

The data for enmetazobactam are consistent with the observations reported by Shapiro *et al*. for sulbactam, who concluded that low inactivation efficiency as well as high turnover rates characterize sulbactam as a poor inhibitor and an efficient substrate for OXA-type β-lactamases (25). Given the strong similarity between enmetazobactam and sulbactam regarding their kinetic behavior toward OXA-23, it can be inferred that enmetazobactam will also function poorly as an inhibitor and will be preferentially hydrolyzed by these enzymes.

Baseline expression levels of OXA-type β-lactamases should therefore be sufficient to effectively hydrolyze enmetazobactam and decrease its antibacterial activity. In this study, we demonstrate that enmetazobactam is a poor inhibitor of *A. baumannii* CHDLs and, importantly, acts as a substrate for these enzymes, undergoing enzymatic hydrolysis. This finding, not previously reported, provides direct evidence that the reduced susceptibility to enmetazobactam in *A. baumannii* is primarily driven by the activity of OXA-type carbapenemases. Consequently, as we have demonstrated above, co-administration of a class D β-lactamase inhibitor with minimal susceptibility to enzymatic degradation, such as durlobactam, can restore the *in vitro* activity of enmetazobactam against multidrug-resistant CRAB.

### Substrate-assisted catalysis as a key mechanism in enmetazobactam hydrolysis by OXA-23

To elucidate the molecular determinants underlying enmetazobactam’s binding affinity and its short residence time, the dynamic behavior of the enmetazobactam/OXA-23 enzyme complex (Michaelis) and its acyl-enzyme adduct in water were explored by Molecular Dynamics (MD) simulations using Amber. Specifically, the aim of conducting these *in silico* studies was to provide some insight into the atomic detail on the ligand binding interactions and conformation of the hydrolytic water molecule responsible for the de-acylation step, as well as its relative allocation to the acyl-serine residue for subsequent nucleophilic attack. Such studies can provide information that cannot be obtained using crystallographic techniques, as the events are transient. The Michaelis complex (enmetazobactam/OXA-23) and its acyl-enzyme adduct were first obtained by docking (no-covalent and covalent, respectively) using the GOLD program and the enzyme coordinates observed in PDBs ID 9NSW and 4JF4. The resulting enzyme complex and adduct were immersed in a truncated octahedron of water molecules and were then subjected to 100 ns of dynamic simulation.

**Fig 3:**
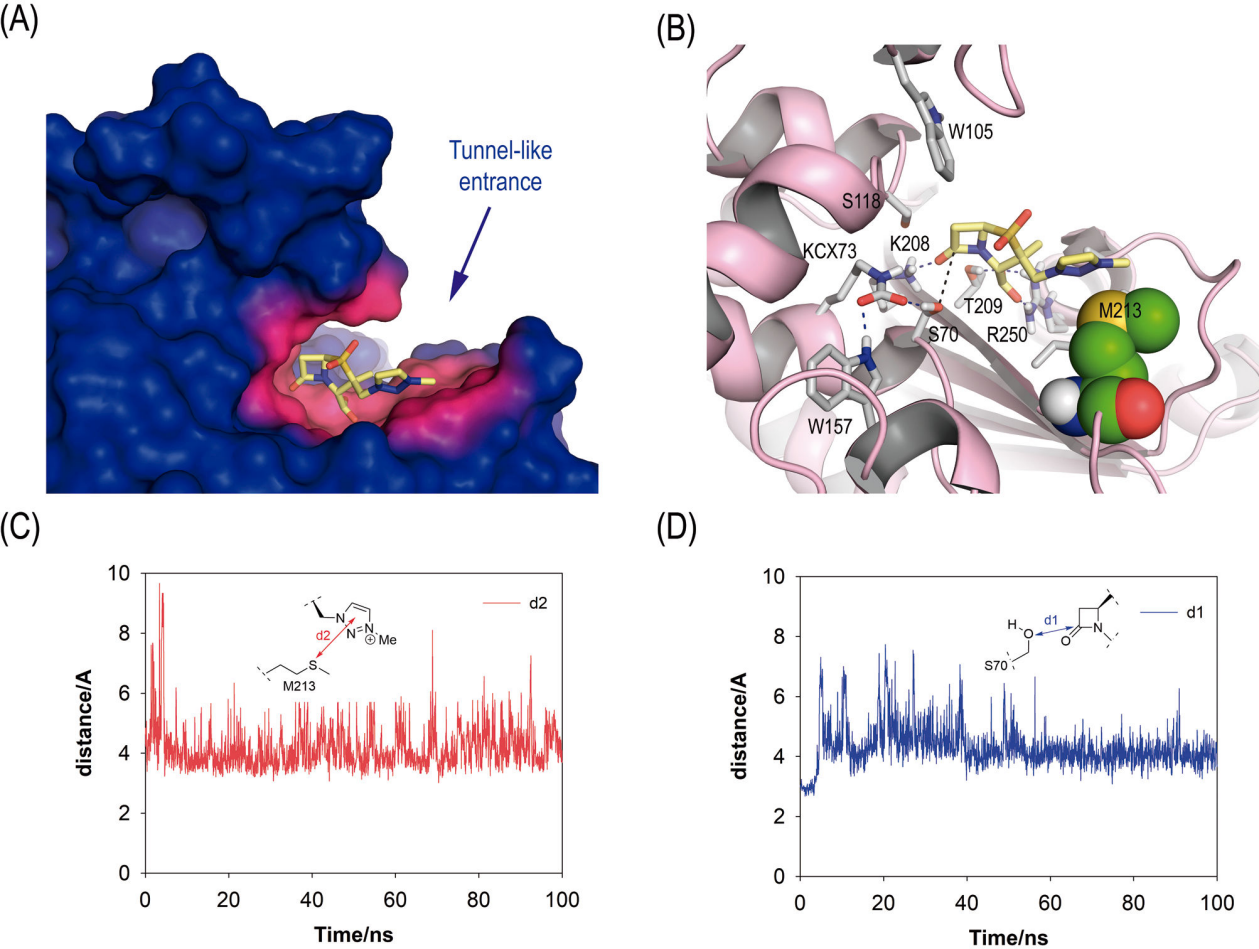
(**A**) Overall view of the three-dimensional structure of the enmetazobactam/OXA-23 Michaelis complex obtained from MD simulations. A snapshot taken after 100 ns of simulation is shown. The enzyme surface is shown in blue, with the region closest to enmetazobactam (ETZ, sticks, yellow) highlighted in pink. (**B**) Key interactions between ETZ and active site residues. Relevant hydrogen-bonding interactions are depicted as blue dashed lines, and key residues are shown and labeled. The distance between the nucleophilic oxygen atom of the catalytic serine (S70) and the carbon atom of the β-lactam ring is shown as a black dashed line. Residue M213, which contributes to the tunnel-like architecture of the OXA-23 active site, is shown as spheres. (**C**) Time evolution of the distance between the sulfur atom of residue M213 and the center of mass of the triazole ring in the Michaelis complex throughout the whole simulation. The average distance was 4.2 Å. (**D**) Time evolution of the distance between the oxygen atom of the catalytic serine residue (S70) and the carbon atom (C7) in the β-lactam ring in the Michaelis complex throughout the simulation. The average distance was 4.3 Å.

The *in silico* outcomes on the Michaelis complex revealed that enmetazobactam would anchor to the active site through strong hydrogen-bonding interactions involving residues R250, T209, and K208, mediated by its carboxylate and carbonyl groups (Fig. 3A and B). Additionally, the arrangement of the ligand in the active site would be stabilized by a strong interaction between the sulfur atom of residue M213, which contributes to the tunnel-like architecture of the OXA-23 active site, and its triazole ring, showing an average distance during the whole simulation of 4.2 Å (Fig. 3C). As a result, the carbonyl group of the ligand would remain near the catalytic serine residue, maintaining a favorable orientation for the subsequent nucleophilic attack. This is supported by the analysis of the evolution of the distance between the involved atoms over the course of the simulation, as well as by the root-mean-square deviation values for the enzyme backbone atoms and enmetazobactam, calculated from the MD simulations of the enmetazobactam/OXA-23 Michaelis complex (Fig. 3D; Fig. S3). On the contrary, simulation studies performed with the sulbactam/OXA-23 Michaelis complex revealed increased ligand mobility within the active site (Fig. S4). We observed that the ligand maintained an optimal arrangement for nucleophilic attack by the catalytic serine residue only during the first 20 ns of the simulation. These results align with the higher *K*_m_ value for sulbactam relative to enmetazobactam, reflecting its lower binding affinity.

**Fig 4:**
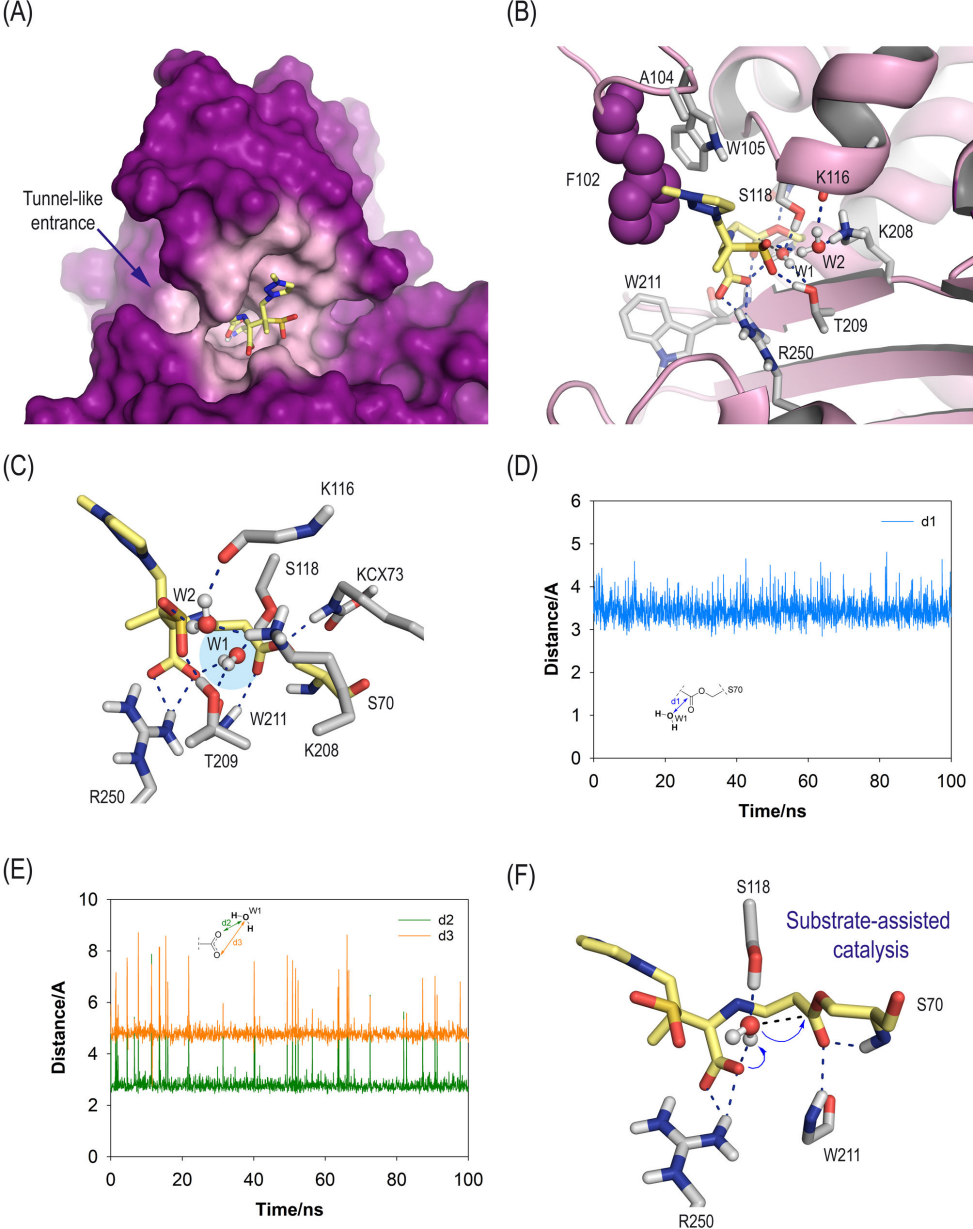
(**A**) Overall view of the three-dimensional structure of the enmetazobactam/OXA-23 acyl-enzyme adduct obtained by MD simulation studies. A snapshot taken after 100 ns of simulation is shown. The region of the enzyme (surface, magenta) closest to the modified enmetazobactam (ETZ, sticks, yellow) is shown in light pink. (**B**) Detailed view of the main contacts with the residues of the active site and key water molecules. Relevant hydrogen-bonding interactions (blue dashed lines) and key residues are shown and labeled. Note that the arrangement of the modified enmetazobactam is frozen by diverse polar interactions as well as by a strong anion-π interaction between the triazole moiety and the essential residue F102 (spheres), which is involved in the enzyme tunnel-like entrance. Under this arrangement, a water molecule (hydrolytic water molecule, W1) with its oxygen’s lone pairs pointing toward the carboxylate group of the modified catalytic serine residue is stabilized during most of the simulation. (**C**) Contacts of the hydrolytic water molecule (W1, blue shading) for turnover, as well as of the bridging water molecule W2 involved in the interaction of the ligand with ε-amino group in residue K208. (**D**) Variation in the relative distances between the carboxylate group (C7 atom) in modified S70 and the hydrolytic water molecule (O atom). (**E**) Variation in the relative distances between the carboxylate group (O2A and O2B atoms) in modified S70 and the hydrolytic water molecule (O atom). (**F**) Proposed substrate-assisted catalysis for turnover, which would explain the experimentally observed short residence time *t*_1/2_ of enmetazobactam.

Our simulation studies involving the enmetazobactam/OXA-23 enzyme adduct revealed that the position of the triazole moiety in the acyl-serine residue would be frozen perpendicular to the side chain of residue F102 with its nitrogen atoms pointing toward the phenyl ring, thus interacting with the tunnel-like entrance characteristic of the OXA-23 enzyme (Fig. 4A and B; Fig. S5). This arrangement would be facilitated by a set of strong hydrogen-bonding interactions with residues R250 and T209, the oxyanion hole (main NH amide groups in W211 and S70), and residues K208 and K116, both mediated by a water molecule (W2). The overall arrangement of the acyl-serine residue would allow the entrapment of a water molecule (W1) in the vicinity of the acyl group (Fig. 4C). W1 would remain frozen in this position for most of the simulation, with one of the oxygen’s lone pairs in an excellent disposition for subsequent nucleophilic attack to the carboxylate group (Fig. S6). Specifically, the hydrogen atoms in W1 would interact by hydrogen bonding with T209 side chain and the carboxylate group in the acyl-serine residue, and one of the oxygen’s lone pairs would interact with S118 side chain (Fig. 4D and E). These findings suggest that the de-acylation step would involve substrate-assisted catalysis in which the carboxylate group in enmetazobactam would act as general base (Fig. 4F) (28). This intramolecular process would increase the reaction rate, thus justifying the experimentally observed short residence time of enmetazobactam.

**Fig 5:**
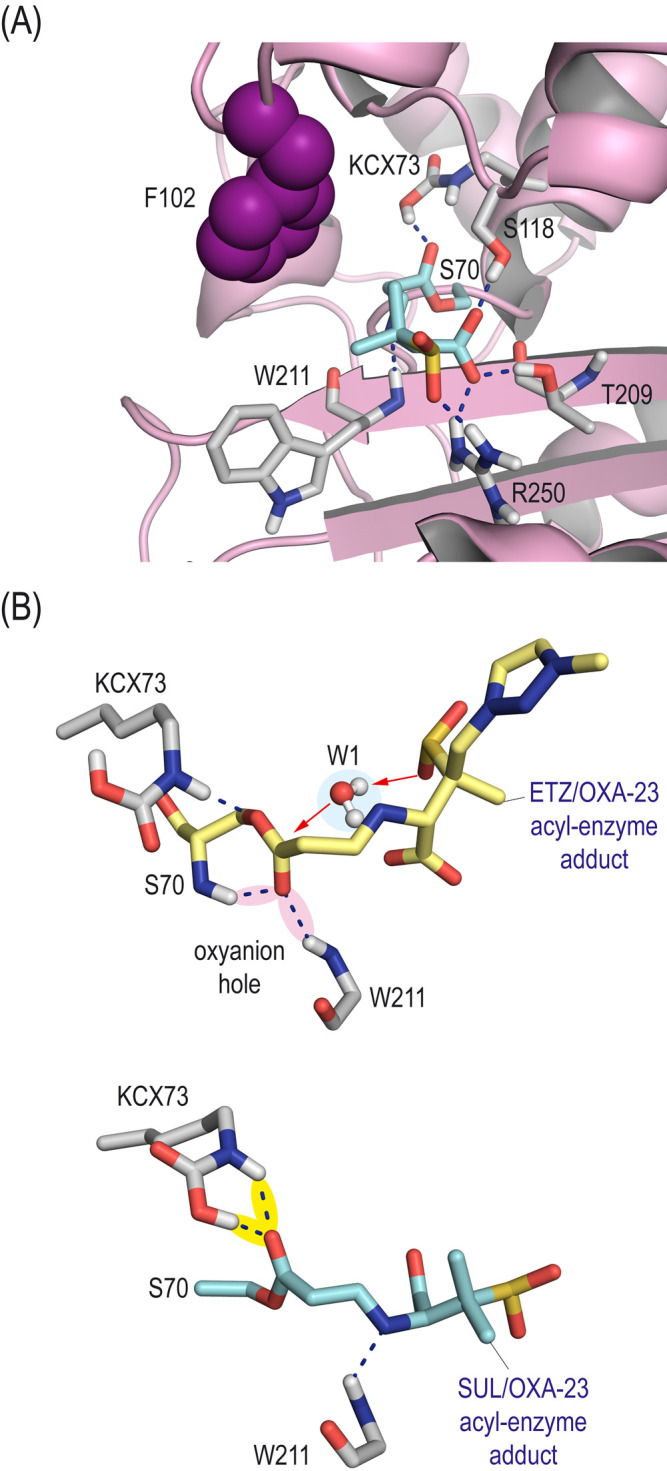
(**A**) Detailed view of OXA-23 enzyme covalently modified by sulbactam (SUL, cyan) obtained by MD simulation studies. A snapshot taken after 100 ns of simulation is shown. Relevant hydrogen-bonding interactions (blue dashed lines) and key residues are shown and labeled. The essential residue F102 is shown in spheres (magenta). (**B**) Comparison of the modified catalytic serine residue S70 by enmetazobactam (green) and sulbactam (cyan) in the corresponding OXA-23 acyl-enzyme adducts. Snapshots taken after 100 ns of simulation are shown. Note that in the ETZ/OXA-23 adduct, the carboxylate group would be activated for hydrolysis by strong hydrogen-bonding interactions with the oxyanion hole (pink shading), and the hydrolytic water molecule is trapped in an appropriate arrangement for nucleophilic attack. By contrast, for the SUL/OXA-23 adduct, oxyanion hole activation of the ester group would not occur as a 180° inversion of this moiety was observed to interact mainly with the carboxylated lysine residue (yellow shading).

For the sulbactam/OXA-23 acyl-enzyme adduct, our computational studies revealed a different scenario (Fig. 5). Specifically, (i) the position of the resulting acyl-serine residue would be less frozen than that of enmetazobactam, since greater motion was observed (Fig. S7); (ii) the arrangement of the acyl-serine residue side chain would be rotated 180° to that of enmetazobactam, thus avoiding the interaction with the oxyanion hole; (iii) no relevant contacts with the tunnel-like entrance were identified; and (iv) no stabilization of the hydrolytic water was observed during the simulation. Therefore, as substrate-assisted catalysis would not occur, the de-acylation step would be slower.

### Conclusion

In conclusion, this study identifies enmetazobactam as a novel β-lactamase inhibitor with intrinsic bactericidal activity against *Acinetobacter* spp., primarily through its strong affinity for PBP2 and PBP3. The reduced susceptibility to enmetazobactam in *A. baumannii* is primarily mediated by OXA-type carbapenemases through substrate-assisted catalysis. Nevertheless, the addition of a class D β-lactamase inhibitor with low vulnerability to enzymatic degradation, such as durlobactam, can effectively restore the antibacterial activity of enmetazobactam against CHDL-producing multidrug-resistant *A. baumannii*. Of relevance, both enmetazobactam and durlobactam have already obtained regulatory approval for clinical use, having met the necessary standards of safety and efficacy, thus positioning the combination as a promising therapeutic candidate for clinical implementation.

To our knowledge, this is the first study to evaluate and report the *in vitro* bactericidal properties of enmetazobactam against *Acinetobacter* spp., thereby contributing novel and clinically relevant insights into its antimicrobial potential. Although further preclinical and clinical studies are warranted to evaluate its pharmacokinetics and *in vivo* activity, the enmetazobactam/durlobactam combination emerges as a promising therapeutic alternative for treating infections caused by CHDL-producing MDR *A. baumannii*, for which treatment options are very limited.

## MATERIALS AND METHODS

### Bacterial collections

The bacterial strains used in this study comprised 208 clinical isolates from the Spanish National *Acinetobacter* spp. 2020 Multicenter Collection, which was previously characterized in detail by our research group, including whole-genome sequencing, phylogenetics studies, clonal dynamics, and resistance mechanisms (2, 29). Additionally, a collection of *A. baumannii* ATCC 17978 transformants coding the *bla*_OXA-23_, *bla*_OXA-24/40_, *bla*_OXA-51_, *bla*_OXA-58_, *bla*_OXA-143_, and *bla*_OXA-201_ genes, previously constructed and described by our group (30), was used to determine the activity of enmetazobactam against strains carrying the main CHDLs of *A. baumannii*.

### MIC, MBC, and synergy testing

The antimicrobial susceptibility of the 208 *Acinetobacter* spp. isolates was assessed using the broth microdilution method, as described in the European Committee on Antimicrobial Susceptibility Testing (EUCAST) v.15 guidelines (http://www.eucast.org/clinical_breakpoints). MICs of cefepime, sulbactam, and imipenem, alone and in combination with enmetazobactam (fixed concentration of 8 mg/L), were determined. MICs for enmetazobactam alone were assessed as well. Similarly, the MICs of both enmetazobactam and sulbactam (for comparison), alone and in combination with durlobactam (fixed concentration of 4 mg/L), were established for the carbapenem-resistant isolates collection. Subsequently, MIC testing was also conducted with a collection of *A. baumannii* ATCC 17978 transformants expressing clinically relevant OXA-like carbapenemase genes.

In order to determine the bactericidal activity of enmetazobactam, MBC testing was carried out by subculturing 100 μL aliquots from wells containing antimicrobial concentrations equal to or greater than the MIC of enmetazobactam onto Mueller-Hinton agar plates. Sulbactam was used as a comparator, as it is a chemically related penicillin-based sulfone inhibitor that also shows intrinsic antibacterial activity against *A. baumannii*. The MBCs were evaluated for a total of nine clinical isolates: (i) three CRAB, (ii) three carbapenem-susceptible *A. baumannii*, and (iii) three *A*. non-*baumannii* isolates. The plates were incubated at 37°C for 24 h, and viable colonies were counted. The MBC was defined as the lowest concentration that resulted in a ≥99.9% reduction of the initial inoculum (31).

Checkerboard assays were performed to assess synergistic interactions between the cefepime/enmetazobactam combination and sulbactam, as well as between enmetazobactam alone and either sulbactam or colistin. These compounds were chosen as the approach suggested by the Infectious Diseases Society of America for the treatment of infections caused by CRAB (32). The assays were conducted with three multidrug-resistant *A. baumannii* isolates from the Spanish Multicenter Collection, each producing a different OXA-type carbapenemase: OXA-23, OXA-24/40, or OXA-58. The FIC index was calculated and interpreted as follows: FIC_index_ ≤ 0.5, synergy; FIC_index_ > 0.5–4, no synergy; and FIC_index_ > 4, antagonism (33, 34).

The *A. baumannii* ATCC 19606 strain was used as the reference strain in all susceptibility experiments, in duplicate assays. Antibiotics were purchased from Sigma-Aldrich, except enmetazobactam and durlobactam, which were obtained from Advanz Pharma and MedChemExpress, respectively.

### PBP-binding assays

The possible involvement of *A. baumannii* PBPs as enmetazobactam targets was evaluated. Thus, the capacity of enmetazobactam and sulbactam (comparator) to bind to PBPs, reported as the 50% inhibitory concentration (IC_50_), was assessed in the wild-type reference *A. baumannii* strain ATCC 19606. The PBP-binding IC_50_ was determined in membrane preparations from three independent experiments, following previously described protocols (13).

Briefly, *A. baumannii* cultures were grown in Mueller-Hinton broth at 37°C with shaking at 180 rpm, and 400 mL were collected in late log phase (OD₆₀₀_nm_= 1), washed, and resuspended in 20 mM KH₂PO₄ and 140 mM NaCl (pH 7.5). After sonication, bacterial membranes were isolated by ultracentrifugation (150,000 *g*, 30 min). The binding assays were conducted with 3.75 µg of membrane proteins, which were incubated for 30 min at 37°C with either enmetazobactam or sulbactam, at concentrations ranging from approximately 0.5 to 256 mg/L. Subsequently, the proteins were labeled with Bocillin FL (Invitrogen, 25 µM), a fluorescent penicillin V analog that reacts with the catalytic serine residue in PBPs. The Bocillin FL signal is inversely proportional to the binding capacity of the compounds tested to PBPs (35). The PBPs were separated by SDS-PAGE (Bio-Rad) and visualized with Odyssey M Imager (LI-COR). The IC₅₀ values were calculated using Empiria Studio software (LI-COR). The binding affinity was determined by comparing the change of the fluorescence in the presence or absence of the tested sulfones.

In order to confirm the involvement of PBPs as targets of enmetazobactam, cell morphology assays involving exposure to enmetazobactam and sulbactam were performed following the methodology described in Supplementary materials and methods.

### Kinetic parameters determination of class D carbapenemase OXA-23 activity against enmetazobactam

One of the most prevalent carbapenemases in *A. baumannii* is OXA-23, a class D β-lactamase (36). Thus, the hydrolytic activity OXA-23 against enmetazobactam and sulbactam was determined using direct spectrophotometric assays. *bla*_OXA-23_ was cloned in the p-GEX-6P-1 plasmid in *Escherichia coli* BL21 and subsequently induced and purified by affinity chromatography using the GST Gene Fusion System (GE Healthcare), following the manufacturer’s instructions. Cloning and purification procedures have previously been described by our research group (37).

OXA-23-catalyzed hydrolysis of enmetazobactam and sulbactam was assayed by monitoring the UV absorbance at 230 nm in a Specord 200 plus spectrophotometer (Analytik Jena), with 0.1 cm path-length quartz cuvettes. All kinetics measurements were performed in triplicate, at 25°C, in 20 mM NaHCO_3_ and 50 mM Na_2_HPO_4_ at pH 7.4. Molar extinction coefficients of 4,999.6 M^−1^cm^−1^ and 1,258.5 M^−1^cm^−1^ were used for enmetazobactam and sulbactam, respectively. To determine these coefficients, the wavelength of maximum absorbance was first established, with peaks detected at 230 nm. Absorbance values were then measured over a concentration range of 40–800 μM for both compounds, conditions selected to ensure the chemical stability of the β-lactamase inhibitors and ensure constant absorptivity. Calibration curves plotting the absorbance vs concentration demonstrated linearity within this range, and molar extinction coefficient was then calculated from the slope of the linear regression according to the Beer-Lambert law.

The Michaelis-Menten constant (*K*_m_) was determined by fitting the data to the Michaelis-Menten equation, following established protocols (38). The *k*_cat_ (hydrolysis rate constant of the β-lactamase) was obtained by linearization of the kinetic data (39). To assess the possible inhibitory capacity of enmetazobactam against the CHDLs of *A. baumannii*, specific OXA-23 inhibition kinetics assays were performed in a Nanoquant Infinite spectrophotometer (Tecan). Specifically, the IC₅₀ was determined as the inhibitor concentration that reduced nitrocefin hydrolysis by 50%, after a 10 min preincubation of the OXA-23 enzyme at room temperature with enmetazobactam, sulbactam, and durlobactam as a comparator (as it is known to be an inhibitor of class D β-lactamases of *A. baumannii*), following established protocols (30). Also, the dissociation constant (*k*_off_), which represents the rate at which the enzyme-inhibitor complex dissociates (i.e., the proportion of the enzyme-inhibitor complex that dissociates per unit of time), was determined through a jump-dilution assay that monitored OXA-23 activity recovery as the enzyme-inhibitor complex dissociated. OXA-23 (1 µM) was preincubated for 30 min at 37°C with excess of inhibitors (50× IC₅₀) to ensure full acyl-enzyme complex formation. Subsequently, the mixture was diluted 1:5,000 to allow gradual complex dissociation. For reaction monitoring, diluted samples were combined with nitrocefin at 3× *K*_m_ of OXA-23 for this substrate. Enzymatic activity, reflecting the progressive dissociation of the acyl-enzyme complex, was monitored spectrophotometrically at 490 nm for 8 h. The resulting progress curves were fitted using GraphPad Prism (GraphPad Software, MA, USA) to equation 1 to estimate *k*_off_, where *V*_0_ is the initial, inhibited enzyme velocity; *V*_*s*_ is the steady-state uninhibited enzyme velocity, and *t* is time. Additionally, the inhibitor-enzyme complex residence time (*t*_1/2_), reflecting the time required for 50% dissociation of the bound inhibitor and thereby the effective duration of enzyme inhibition, was calculated from the fitted data using GraphPad Prism, as previously described (37).


(1) $$P=V{}_{s}\;\times t+(V{}_{0}-V{}_{s})\times [1-\exp^{\left(-k_{\text{off}}\;\times \;t\right)}]/k_{\text{off}}$$

### Computational studies

The molecular basis of the binding affinity of enmetazobactam, along with the rapid deacylation process during its OXA-23 catalyzed hydrolysis, was explored *in silico*. To this end, the dynamic behavior of the enmetazobactam/OXA-23 enzyme complex and its acyl-enzyme adduct in a truncated octahedron of TIP3P water molecules was studied by a combination of docking and MD simulations. A procedure similar to that previously described by our research group for the PDC-1 enzyme was used (40). The enzyme coordinates identified in the crystal structure of the wild-type form (PDB ID 9NSW, 1.4 Å) (41) and the meropenem/OXA-23 acyl-enzyme adduct (PDB ID 4JF4, 2.14 Å, chain A) were applied (26). The same procedure was carried out with sulbactam. The standardized numbering protocol for class D enzymes, namely SAND, was used (42). Specific details of the experimental procedure are indicated in the Supplementary materials and methods.

## Data Availability

The data supporting this work are available under NCBI Bioproject PRJNA991768 and PRJNA1045406.

## SUPPLEMENTAL MATERIAL

Supplemental material aac.01206-25-s0001.docx Tables S1 to S7; Fig. S1 to S7.

ASM does not own the copyrights to Supplemental Material that may be linked to, or accessed through, an article. The authors have granted ASM a non-exclusive, world-wide license to publish the Supplemental Material files. Please contact the corresponding author directly for reuse.
