# Supplementary material for: Antibacterial activity of enmetazobactam against Acinetobacter spp.: a molecular dissection of mechanism of action and resistance determinants
Source: Antimicrob Agents Chemother. 2025 Dec 22;70(2):e01206-25. doi: 10.1128/aac.01206-25 (PMC12888861; doi:10.1128/aac.01206-25)
Supplement: Supplemental material — Tables S1 to S7; Fig. S1 to S7. [file aac.01206-25-s0001.docx]

**Supplementary Material**

**Title:**

**Antibacterial activity of enmetazobactam against *Acinetobacter* spp*.*: a molecular dissection of mechanism of action and resistance determinants**

**Authors:** Gabriela-Alejandra Báez-Barroso, Arianna Rodríguez-Coello, Juan Carlos Vázquez-Ucha, Silvia López-Argüello, Michelle Outeda-García, Lucía González-Pinto, Andrea García-Pose, Paula Guijarro-Sánchez, Isaac Alonso-García, Emilio Lence, Concepción González-Bello, Antonio Oliver, Jorge Arca-Suárez, Bartolome Moya, Germán Bou, Alejandro Beceiro on behalf of the Spanish National *Acinetobacter* spp. 2020 Study Group.

**Supplementary Material and Methods**______________Page 2

**References______________________________________**Page 3

**Supplementary Table 1**____________________________Page 5

**Supplementary Table 2**____________________________Page 8

**Supplementary Table 3**____________________________Page 10

**Supplementary Table 4**____________________________Page 14

**Supplementary Table 5**____________________________Page 15

**Supplementary Table 6**____________________________Page 16

**Supplementary Table 7**____________________________Page 18

**Supplementary Figure 1**___________________________Page 19

**Supplementary Figure 2**___________________________Page 20

**Supplementary Figure 3**___________________________Page 21

**Supplementary Figure 4**___________________________Page 22

**Supplementary Figure 5**___________________________Page 23

**Supplementary Figure 6**___________________________Page 24

**Supplementary Figure 7**___________________________Page 25

**SUPPLEMENTARY MATERIAL AND METHODS**

**Evaluation of PBPs as targets by morphological analysis in *A. baumannii***

To confirm the involvement of PBPs as targets of enmetazobactam, the cellular morphology of *A. baumannii* ATCC 19606 reference strain was analyzed after exposure to the compound at different concentrations. Bright-field microscopy was used to monitor the cell morphology. The strain was cultured overnight in Mueller-Hinton broth at 37 °C with agitation (150 rpm) until reaching an approximate OD_600nm_ of 1, in the presence of enmetazobactam at concentrations of 0.5x, 1x, and 2x the MIC. Sulbactam and imipenem were included as positive controls at the same concentrations relative to their respective MICs, as they exhibit known activity mainly against PBP2 (imipenem) and PBP3 (sulbactam) in  *A. baumannii*. For microscopic visualization, aliquots of each sample were obtained, stained with crystal violet solution and observed using an Olympus BX61 microscope (Olympus).

Changes in cell morphology were determined on the basis of alterations in septal integrity, abnormal elongation, spheroplast formation and filamentation, patterns previously associated with the selective inhibition of PBP1, PBP2 and PBP3 in Gram-negative bacilli. Specifically, PBP1 inhibition has been linked to cell lysis, PBP2 inhibition has been linked to spherification, and PBP3 inhibition to defective cell division and filament formation (1, 2). The results were qualitatively compared across different treatments to infer the potential selectivity of enmetazobactam for specific PBPs.

**Computational studies**

Docking and Molecular Dynamics (MD) simulation studies were carried out following our previously reported protocol with the PDC-1 enzyme (3). Version 2024.3.0 of GOLD software (4) and the enzyme coordinates identified in the crystal structure of the meropenem/OXA-23 acyl-enzyme adduct (PDB ID 4JF4 (5), 2.14 Å, chain A) and its apo-form (PDB ID 9NSW (6), 1.40 Å) were used. The protonation state of titratable groups at pH 7.0 was determined using the H^++^ Web server (7,8). As a result of this analysis, residues H28 and H32 were protonated in the ε position and H178 in δ. K73 was considered in the carboxylated form (KCX73). The covalently modified serine residue with enmetazobactam and sulbactam were parameterized using the R.E.D. server (version III.5x), by adding acetyl and *N*-methyl caps and applying the same procedure used for ligands (9–11). The simulation time was 100 ns. These studies were conducted using the Amber20 and AmberTools21 suite of programs (12). The enzyme structures were analyzed and represented using the program PyMOL (13). The trajectories and root-mean-square deviation (rmsd) throughout the whole simulation were calculated using the cpptraj module in Amber20 (14). The standardized numbering protocol for class D enzymes, namely SAND, was employed (15).

**References**:

1. Buijs J, Dofferhoff ASM, Mouton JW, Wagenvoort JHT, van der Meer JWM. 2008. Concentration-dependency of beta-lactam-induced filament formation in Gram-negative bacteria*. Clin Microbiol Infect* 14:344–349.
2. Sumita Y, Fukasawa M, Okuda T. 1990. Comparison of two carbapenems, SM-7338 and imipenem: affinities for penicillin-binding proteins and morphological changes. *J Antibiot (Tokyo)* 43:314–320.
3. Vázquez-Ucha JC, Rodríguez D, Lasarte-Monterrubio C, Lence E, Arca-Suarez J, Maneiro M, Gato E, Perez A, Martínez-Guitián M, Juan C, Oliver A, Bou G, González-Bello C, Beceiro A. 2021. 6-Halopyridylmethylidene penicillin-based sulfones efficiently inactivate the natural resistance of *Pseudomonas aeruginosa* to β-lactam antibiotics. *J Med Chem* 64:6310–6328.
4. Jones G, Willett P, Glen RC, Leach AR, Taylor R. 1997. Development and validation of a genetic algorithm for flexible docking. *J Mol Biol* 267:727–748.
5. Smith CA, Antunes NT, Stewart NK, Toth M, Kumarasiri M, Chang M, Mobashery S, Vakulenko SB. 2013. Structural basis for carbapenemase activity of the OXA-23 β-lactamase from *Acinetobacter baumannii*. *Chem Biol* 20:1107–1115.
6. Toth M, Stewart NK, Quan P, Khan MMK, Cox J, Buynak JD, Smith CA, Vakulenko SB. 2025. Dual mechanism of the OXA-23 carbapenemase inhibition by the carbapenem NA-1-157. *Antimicrob Agents Chemother* 69:e0091825.
7. Gordon JC, Myers JB, Folta T, Shoja V, Heath LS, Onufriev A. 2005. H++: a server for estimating pKas and adding missing hydrogens to macromolecules. *Nucleic Acids Res* 33(Web Server issue):W368–W371.
8. <http://newbiophysics.cs.vt.edu/H++/> (accessed June 18, 2025).
9. <http://upjv.q4md-forcefieldtools.org/RED> (accessed June 18, 2025).
10. Dupradeau, F.-Y.; Pigache, A.; Zaffran, T.; Savineau, C.; Lelong, R.; Grivel, N.; Lelong, D.; Rosanski, W.; Cieplak, P. 2010. The R.E.D. tools: advances in RESP and ESP charge derivation and force field library building. *Phys Chem Chem Phys* 12:7821–7839.
11. Vanquelef E; Simon S, Marquant G, Garcia E, Klimerak G, Delepine JC, Cieplak P, Dupradeau F-Y. 2011. R.E.D. Server: a web service for deriving RESP and ESP charges and building force field libraries for new molecules and molecular fragments. *Nucleic Acids Res* 39:W511– W517.
12. Case DA, Aktulga HM, Belfon K, Cerutti DS, Cisneros GA, Cruzeiro VWD, Forouzesh N, Giese TJ, Götz AW, Gohlke H, Izadi S, Kasavajhala K, Kaymak MC, King E, Kurtzman T, Lee T-S, Li P, Liu J, Luchko T, Luo R, Manathunga M, Machado MR, Nguyen HM, O’Hearn KA, Onufriev AV, Pan F, Pantano S, Qi R, Rahnamoun A, Risheh A, Schott-Verdugo S, Shajan A, Swails J, Wang J, Wei H, Wu X, Wu Y, Zhang S, Zhao S, Zhu Q, Cheatham III TE, Roe DR, Roitberg A, Simmerling C, York DM, Nagan MC, Merz Jr KM. 2023. AmberTools. *J Chem Inf Model* 63:6183–6191.
13. DeLano WL. 2008. The PyMOL molecular graphics system; DeLano scientific LLC. Palo Alto, CA. Available from: <http://www.pymol.org/> (accessed June 18, 2025).
14. Roe DR, Cheatham TE. 2013. PTRAJ and CPPTRAJ: software for processing and analysis of molecular dynamics trajectory data. *J Chem Theory Comput* 9:3084–3095.
15. Attana F, Kim S, Spencer J, Iorga BI, Docquier J, Rossolini GM, Perilli M, Amicosante G, Vila AJ, Vakulenko SB, Mobashery S, Bradford P, Bush K, Partridge SR, Hujer AM, Hujer KM, Bonomo RA, Haider S. 2025. SAND: a comprehensive annotation of class D β-lactamases using structural alignment-based numbering. *Antimicrob Agents Chemother* 69:e00150-25.

**SUPPLEMENTARY TABLES**

**Supplementary Table 1.** MIC (mg/L) values for the antibiotics tested against the collection of carbapenem-resistant *A. baumannii* isolates (n = 67).

| **Isolate number** | **Species** | **ST** | **β-lactamases** | **ETZ** | **FEP** | **FEP/ETZ** | **IMI** | **IMI/ETZ** | **SUL** | **SUL/ETZ** |
| --- | --- | --- | --- | --- | --- | --- | --- | --- | --- | --- |
| Ab4 | *A. baumannii* | 2 | ADC-30, OXA-66, OXA-23 | 64 | 32 | 32 | 32 | 32 | 16 | 16 |
| Ab5 | *A. baumannii* | 2 | ADC-30, OXA-66, OXA-23 | 64 | 64 | 32 | 32 | 32 | 16 | 16 |
| Ab6 | *A. baumannii* | 2 | ADC-30, OXA-66, IS*Aba1*-OXA-23, TEM-12 | 64 | 16 | 16 | 32 | 16 | 16 | 8 |
| Ab7 | *A. baumannii* | 2 | ADC-30, OXA-66, OXA-23 | 64 | 64 | 32 | 32 | 8 | 32 | 16 |
| Ab8 | *A. baumannii* | 2 | ADC-30, OXA-66, OXA-23 | 64 | 32 | 32 | 32 | 32 | 16 | 16 |
| Ab9 | *A. baumannii* | 2 | ADC-30, OXA-66, OXA-23, TEM-12 | 32 | 32 | 16 | 16 | 8 | 32 | 8 |
| Ab10 | *A. baumannii* | 2 | ADC-30, OXA-66, OXA-23 | 64 | 32 | 32 | 32 | 32 | 16 | 16 |
| Ab12 | *A. baumannii* | 2 | ADC-30, IS*Aba1*-OXA-201 | 4 | 8 | ≤0.06 | 8 | ≤0.06 | 2 | ≤0.06 |
| Ab13 | *A. baumannii* | 2 | ADC-30, IS*Aba1*-OXA-201 | 4 | 16 | ≤0.06 | 8 | ≤0.06 | 2 | ≤0.06 |
| Ab14 | *A. baumannii* | 2 | ADC-30, IS*Aba1*-OXA-201 | 4 | 16 | ≤0.06 | 16 | ≤0.06 | 2 | ≤0.06 |
| Ab15 | *A. baumannii* | 2 | ADC-30, IS*Aba1*-OXA-201 | 8 | 16 | ≤0.06 | 16 | ≤0.06 | 2 | ≤0.06 |
| Ab16 | *A. baumannii* | 2 | ADC-30, IS*Aba1*-OXA-201 | 8 | 16 | ≤0.06 | 8 | ≤0.06 | 2 | ≤0.06 |
| Ab17 | *A. baumannii* | 2 | ADC-30, IS*Aba1*-OXA-201 | 8 | 16 | ≤0.06 | 16 | ≤0.06 | 2 | ≤0.06 |
| Ab18 | *A. baumannii* | 2 | ADC-30, IS*Aba1*-OXA-201 | 4 | 16 | ≤0.06 | 8 | ≤0.06 | 2 | ≤0.06 |
| Ab19 | *A. baumannii* | 2 | ADC-30, OXA-66, OXA-58 | 16 | 64 | 8 | 32 | 16 | 8 | 8 |
| Ab20 | *A. baumannii* | 2 | ADC-30, IS*Aba1*-OXA-201 | 4 | 16 | ≤0.06 | 8 | ≤0.06 | 2 | ≤0.06 |
| Ab24 | *A. baumannii* | 2 | ADC-30, IS*Aba1*-OXA-201 | 4 | 16 | ≤0.06 | 16 | ≤0.06 | 2 | ≤0.06 |
| Ab25 | *A. baumannii* | 2 | ADC-30, IS*Aba1*-OXA-201 | 4 | 32 | ≤0.06 | 16 | ≤0.06 | 2 | ≤0.06 |
| Ab26 | *A. baumannii* | 2 | ADC-30, OXA-66, OXA-58 | 16 | 64 | 16 | 8 | 8 | 16 | 2 |
| Ab28 | *A. baumannii* | 2 | IS*Aba1*-ADC-30, OXA-66, OXA-23 | 64 | 64 | 32 | 32 | 16 | 16 | 16 |
| Ab29 | *A. baumannii* | 2 | ADC-30, OXA-66, OXA-23 | 64 | 32 | 32 | 64 | 32 | 32 | 16 |
| Ab30 | *A. baumannii* | 2 | IS*Aba1*-ADC-30, OXA-66, OXA-23 | 64 | 32 | 16 | 64 | 64 | 16 | 16 |
| Ab31 | *A. baumannii* | 2 | ADC-30, OXA-66, OXA-23 | 64 | 32 | 16 | 64 | 32 | 16 | 16 |
| Ab32 | *A. baumannii* | 2 | IS*Aba1*-ADC-30, OXA-66, OXA-23-23 | 64 | 32 | 16 | 64 | 32 | 16 | 16 |
| Ab33 | *A. baumannii* | 2 | ADC-30, OXA-66, OXA-23 | 64 | 64 | 32 | 64 | 64 | 16 | 16 |
| Ab35 | *A. baumannii* | 2 | ADC-30, OXA-66, OXA-23 | 64 | 32 | 16 | 32 | 16 | 16 | 8 |
| Ab36 | *A. baumannii* | 2 | ADC-30, OXA-66, OXA-23 | 64 | 64 | 32 | 64 | 64 | 16 | 16 |
| Ab37 | *A. baumannii* | 2 | ADC-30, OXA-66, OXA-23 | 64 | 32 | 32 | 64 | 64 | 16 | 16 |
| Ab39 | *A. baumannii* | 2 | ADC-30, OXA-66, OXA-23 | 16 | 4 | 2 | 16 | 4 | 4 | 1 |
| Ab41 | *A. baumannii* | 2 | ADC-30, OXA-66, OXA-23 | 64 | 64 | 32 | 64 | 64 | 16 | 16 |
| Ab42 | *A. baumannii* | 2 | ADC-30, OXA-66, OXA-23 | 64 | 32 | 32 | 64 | 64 | 16 | 16 |
| Ab44 | *A. baumannii* | 2 | ADC-30, OXA-66, OXA-23 | 32 | 32 | 16 | 32 | 16 | 8 | 8 |
| Ab45 | *A. baumannii* | 1 | ADC-75, OXA-69, OXA-23 | 64 | 64 | 32 | 32 | 16 | 16 | 8 |
| Ab46 | *A. baumannii* | 1 | ADC-75, OXA-69, OXA-23 | 64 | 64 | 32 | 32 | 32 | 16 | 16 |
| Ab47 | *A. baumannii* | 1 | ADC-75, OXA-69, OXA-23 | 64 | 64 | 32 | 32 | 32 | 16 | 8 |
| Ab49 | *A. baumannii* | 1 | ADC-75, OXA-69, OXA-23 | 64 | 64 | 32 | 64 | 32 | 16 | 8 |
| Ab52 | *A. baumannii* | 1 | ADC-75, OXA-69, OXA-23 | 64 | 32 | 32 | 16 | 16 | 8 | 8 |
| Ab53 | *A. baumannii* | 1 | IS*Aba1*-ADC-75, OXA-69, OXA-23 | 64 | 32 | 32 | 32 | 32 | 16 | 8 |
| Ab54 | *A. baumannii* | 1 | ADC-75, OXA-69, OXA-23 | 64 | 64 | 32 | 32 | 32 | 16 | 8 |
| Ab55 | *A. baumannii* | 1 | ADC-75, OXA-69, OXA-23 | 64 | 32 | 32 | 32 | 32 | 8 | 8 |
| Ab56 | *A. baumannii* | 1 | ADC-75, OXA-69, OXA-23 | 64 | 32 | 32 | 16 | 16 | 8 | 8 |
| Ab83 | *A. baumannii* | 25 | ADC-5, OXA-64, OXA-23 | 64 | 64 | 32 | 32 | 32 | 32 | 16 |
| Ab109 | *A. baumannii* | 1 | ADC-75, OXA-69, OXA-23 | 32 | 64 | 32 | 16 | 16 | 16 | 8 |
| Ab133 | *A. baumannii* | 2 | ADC-30, OXA-66, OXA-58, TEM-12 | 8 | 32 | 8 | 16 | 16 | 32 | 8 |
| Ab134 | *A. baumannii* | 2 | ADC-30, OXA-66, OXA-58, TEM-12 | 8 | 16 | 8 | 16 | 8 | 32 | 4 |
| Ab137 | *A. baumannii* | 85 | ADC-2, OXA-94, OXA-23 | 64 | 64 | 32 | 32 | 32 | 16 | 16 |
| Ab138 | *A. baumannii* | 745 | ADC-30, OXA-66, OXA-58 | 64 | 32 | 16 | 32 | 16 | 8 | 4 |
| Ab140 | *A. baumannii* | 85 | ADC-2, OXA-94, OXA-23 | 64 | 32 | 32 | 32 | 32 | 16 | 16 |
| Ab141 | *A. baumannii* | 745 | ADC-30, OXA-66, OXA-58 | 8 | 32 | 8 | 16 | 8 | 8 | 4 |
| Ab144 | *A. baumannii* | 745 | ADC-30, OXA-66, OXA-58 | 32 | 32 | 8 | 32 | 16 | 8 | 4 |
| Ab169 | *A. baumannii* | 2 | ADC-30, OXA-66, OXA-23 | 64 | 32 | 16 | 32 | 16 | 16 | 8 |
| Ab171 | *A. baumannii* | 2 | ADC-30, OXA-66, OXA-23 | 64 | 32 | 32 | 16 | 16 | 16 | 16 |
| Ab173 | *A. baumannii* | 2 | IS*Aba1*-ADC-30, OXA-66, OXA-23 | 64 | ≥128 | 64 | 32 | 16 | 32 | 32 |
| Ab174 | *A. baumannii* | 2 | ADC-30, OXA-66, OXA-23 | 64 | 32 | 32 | 64 | 32 | 16 | 16 |
| Ab179 | *A. baumannii* | 2 | ADC-30, OXA-66, OXA-23 | 64 | 64 | 64 | 32 | 32 | 16 | 16 |
| Ab180 | *A. baumannii* | 2 | ADC-30, OXA-66, OXA-24/40 | 32 | 16 | 8 | 64 | 32 | 4 | 4 |
| Ab181 | *A. baumannii* | 2 | ADC-30, OXA-66, OXA-23 | 32 | 32 | 32 | 32 | 16 | 16 | 16 |
| Ab182 | *A. baumannii* | 2 | ADC-30, OXA-66, OXA-23 | 16 | 16 | 4 | 16 | 2 | 8 | 4 |
| Ab183 | *A. baumannii* | 2 | ADC-30, OXA-66, OXA-23 | 32 | 32 | 16 | 16 | 16 | 8 | 8 |
| Ab184 | *A. baumannii* | 2 | ADC-30, OXA-66, OXA-23 | 32 | ≥128 | 64 | 32 | 16 | 16 | 16 |
| Ab186 | *A. baumannii* | 2 | ADC-30, OXA-66, OXA-23 | 32 | 32 | 32 | 32 | 16 | 16 | 16 |
| Ab191 | *A. baumannii* | 2 | ADC-30, OXA-66, OXA-23 | 64 | 32 | 32 | 64 | 32 | 16 | 8 |
| Ab192 | *A. baumannii* | 2 | ADC-30, OXA-66, OXA-23 | 64 | 32 | 32 | 64 | 32 | 16 | 8 |
| Ab194 | *A. baumannii* | 2 | ADC-30, OXA-66, OXA-23 | 64 | 32 | 32 | 32 | 16 | 16 | 8 |
| Ab199 | *A. baumannii* | 2 | ADC-30, OXA-66, OXA-23 | 32 | 32 | 16 | 32 | 16 | 16 | 8 |
| Ab201 | *A. baumannii* | 2 | ADC-30, OXA-66, OXA-23 | 64 | 64 | 32 | 32 | 16 | 16 | 4 |
| Ab204 | *A. baumannii* | 2 | ADC-30, OXA-66, OXA-23 | 32 | 16 | 8 | 16 | 8 | 8 | 8 |

Enmetazobactam was used at a fixed concentration of 8 mg/L. ETZ, enmetazobactam; FEP, cefepime; IMI, imipenem, SUL, sulbactam.

**Supplementary Table 2.** MIC (mg/L) values for the antibiotics tested against the collection of carbapenem-susceptible *A. baumannii* isolates (n = 54).

| **Isolate number** | **Species** | **ST** | **β-lactamases** | **ETZ** | **FEP** | **FEP/ETZ** | **IMI** | **IMI/ETZ** | **SUL** | **SUL/ETZ** |
| --- | --- | --- | --- | --- | --- | --- | --- | --- | --- | --- |
| Ab1 | *A. baumannii* | 164 | ADC-6, OXA-91 | 4 | 2 | ≤0.06 | 0.12 | ≤0.06 | 1 | ≤0.06 |
| Ab11 | *A. baumannii* | 239 | ADC-2, OXA-51 | 4 | 2 | ≤0.06 | 0.12 | ≤0.06 | 1 | ≤0.06 |
| Ab34 | *A. baumannii* | 2182 | ADC-2, OXA-130 | 4 | 2 | ≤0.06 | 0.25 | ≤0.06 | 0.5 | ≤0.06 |
| Ab38 | *A. baumannii* | 866 | ADC-2, OXA-385 | 4 | 8 | ≤0.06 | 0.25 | ≤0.06 | 1 | ≤0.06 |
| Ab40 | *A. baumannii* | 2176 | ADC-2, OXA-64 | 4 | 2 | ≤0.06 | 0.25 | ≤0.06 | 1 | ≤0.06 |
| Ab50 | *A. baumannii* | 2176 | ADC-2, OXA-64 | 4 | 2 | ≤0.06 | 0.25 | ≤0.06 | 1 | ≤0.06 |
| Ab57 | *A. baumannii* | 1470 | ADC-58, OXA-106 | 2 | 2 | ≤0.06 | 0.25 | ≤0.06 | 1 | ≤0.06 |
| Ab58 | *A. baumannii* | 6 | ADC-2, OXA-94 | 4 | 4 | ≤0.06 | 0.25 | ≤0.06 | 2 | ≤0.06 |
| Ab61 | *A. baumannii* | 1639 | ADC-7, OXA-64 | 4 | 1 | ≤0.06 | 0.12 | ≤0.06 | 2 | ≤0.06 |
| Ab62 | *A. baumannii* | 265 | ADC-2, OXA-91 | 4 | 1 | ≤0.06 | 0.25 | ≤0.06 | 1 | ≤0.06 |
| Ab67 | *A. baumannii* | 1 | ADC-11, OXA-69 | 4 | 2 | ≤0.06 | 0.25 | ≤0.06 | 1 | ≤0.06 |
| Ab79 | *A. baumannii* | 106 | ADC-11, OXA-78 | 4 | 1 | ≤0.06 | 0.12 | ≤0.06 | 1 | ≤0.06 |
| Ab80 | *A. baumannii* | 2182 | ADC-6, OXA-208 | 4 | 1 | ≤0.06 | 0.12 | ≤0.06 | 4 | ≤0.06 |
| Ab81 | *A. baumannii* | 25 | ADC-79, OXA-64 | 4 | 2 | ≤0.06 | 0.12 | ≤0.06 | 2 | ≤0.06 |
| Ab93 | *A. baumannii* | 25 | ADC-79, OXA-64 | 4 | 2 | ≤0.06 | 0.12 | ≤0.06 | 1 | ≤0.06 |
| Ab95 | *A. baumannii* | 582 | ADC-5, OXA-317 | 2 | 2 | ≤0.06 | 0.12 | ≤0.06 | 1 | ≤0.06 |
| Ab96 | *A. baumannii* | 582 | ADC-5, OXA-317 | 2 | 1 | ≤0.06 | 0.12 | ≤0.06 | 1 | ≤0.06 |
| Ab100 | *A. baumannii* | 145 | ADC-58, OXA-343 | 2 | 2 | ≤0.06 | 0.12 | ≤0.06 | 1 | ≤0.06 |
| Ab110 | *A. baumannii* | 1623 | ADC-3, OXA-70 | 4 | 8 | ≤0.06 | 0.25 | ≤0.06 | 1 | ≤0.06 |
| Ab112 | *A. baumannii* | 193 | ADC-like, OXA-120 | 2 | 0.5 | ≤0.06 | 0.12 | ≤0.06 | 0.5 | ≤0.06 |
| Ab124 | *A. baumannii* | 2176 | ADC-2, OXA-64 | 2 | 1 | ≤0.06 | 0.12 | ≤0.06 | 1 | ≤0.06 |
| Ab127 | *A. baumannii* | 2176 | ADC-2, OXA-64 | 4 | 2 | ≤0.06 | 0.12 | ≤0.06 | 2 | ≤0.06 |
| Ab128 | *A. baumannii* | 2176 | ADC-2, OXA-64 | 2 | 1 | ≤0.06 | 0.12 | ≤0.06 | 1 | ≤0.06 |
| Ab130 | *A. baumannii* | 32 | ADC-79, OXA-100 | 2 | 4 | ≤0.06 | 0.12 | ≤0.06 | 2 | ≤0.06 |
| Ab131 | *A. baumannii* | 687 | ADC-like, OXA-208 | 2 | 0.5 | ≤0.06 | 0.12 | ≤0.06 | 1 | ≤0.06 |
| Ab132 | *A. baumannii* | 1112 | ADC-79, OXA-89 | 2 | 4 | ≤0.06 | 0.12 | ≤0.06 | 2 | ≤0.06 |
| Ab136 | *A. baumannii* | 2183 | ADC-like, OXA-66 | 4 | 2 | ≤0.06 | 0.12 | ≤0.06 | 1 | ≤0.06 |
| Ab139 | *A. baumannii* | 54 | ADC-like, OXA-365 | 4 | 2 | ≤0.06 | 0.25 | ≤0.06 | 1 | ≤0.06 |
| Ab142 | *A. baumannii* | 1 | ADC-like, OXA-69 | 8 | 16 | ≤0.06 | 1 | ≤0.06 | 4 | ≤0.06 |
| Ab143 | *A. baumannii* | 1328 | ADC-76, OXA-408 | 4 | 2 | ≤0.06 | 0.12 | ≤0.06 | 2 | ≤0.06 |
| Ab149 | *A. baumannii* | 1112 | ADC-6, OXA-407 | 8 | 2 | ≤0.06 | 0.25 | ≤0.06 | 1 | ≤0.06 |
| Ab150 | *A. baumannii* | 2184 | ADC-like, OXA-64 | 4 | 2 | ≤0.06 | 0.25 | ≤0.06 | 2 | ≤0.06 |
| Ab152 | *A. baumannii* | 132 | ADC-2, OXA-120 | 4 | 2 | ≤0.06 | 0.25 | ≤0.06 | 1 | ≤0.06 |
| Ab158 | *A. baumannii* | 2034 | ADC-2, OXA-217 | 2 | 0.5 | ≤0.06 | ≤0.06 | ≤0.06 | 0.5 | ≤0.06 |
| Ab163 | *A. baumannii* | 132 | ADC-2, OXA-120 | 2 | 0.5 | ≤0.06 | ≤0.06 | ≤0.06 | 0.5 | ≤0.06 |
| Ab167 | *A. baumannii* | 1623 | ADC-like, OXA-345 | 2 | 1 | ≤0.06 | 0.12 | ≤0.06 | 0.5 | ≤0.06 |
| Ab168 | *A. baumannii* | 1470 | ADC-75, OXA-402 | 2 | 1 | ≤0.06 | 0.25 | ≤0.06 | 0.5 | ≤0.06 |
| Ab170 | *A. baumannii* | 2110 | ADC-4, OXA-338 | 4 | 2 | ≤0.06 | 0.12 | ≤0.06 | 1 | ≤0.06 |
| Ab172 | *A. baumannii* | 2185 | ADC-5, OXA-217 | 2 | 1 | ≤0.06 | ≤0.06 | ≤0.06 | 2 | ≤0.06 |
| Ab185 | *A. baumannii* | 2 | ADC-26, OXA-66 | 4 | 4 | ≤0.06 | 0.25 | ≤0.06 | 1 | ≤0.06 |
| Ab187 | *A. baumannii* | 105 | ADC-2, OXA-430 | 2 | 0.5 | ≤0.06 | ≤0.06 | ≤0.06 | 1 | ≤0.06 |
| Ab188 | *A. baumannii* | 203 | ADC-6, OXA-217, PDC-1 | 4 | 0.5 | ≤0.06 | 0.12 | ≤0.06 | 1 | ≤0.06 |
| Ab189 | *A. baumannii* | 1405 | ADC-3, OXA-120 | 2 | 1 | ≤0.06 | ≤0.06 | ≤0.06 | 0.5 | ≤0.06 |
| Ab190 | *A. baumannii* | 2186 | ADC-6, OXA-413 | 2 | 1 | ≤0.06 | 0.25 | ≤0.06 | 0.5 | ≤0.06 |
| Ab193 | *A. baumannii* | 164 | ADC-6, OXA-91 | 2 | 1 | ≤0.06 | ≤0.06 | ≤0.06 | 0.5 | ≤0.06 |
| Ab195 | *A. baumannii* | 1 | ADC-11, OXA-69 | 2 | 2 | ≤0.06 | 0.12 | ≤0.06 | 1 | ≤0.06 |
| Ab200 | *A. baumannii* | 1336 | ADC-2, OXA-51 | 2 | 2 | ≤0.06 | 0.12 | ≤0.06 | 0.5 | ≤0.06 |
| Ab202 | *A. baumannii* | 1 | ADC-11, OXA-69 | 2 | 2 | ≤0.06 | 0.12 | ≤0.06 | 1 | ≤0.06 |
| Ab203 | *A. baumannii* | 221 | ADC-6, OXA-88 | 2 | 1 | ≤0.06 | 0.12 | ≤0.06 | 0.5 | ≤0.06 |
| Ab206 | *A. baumannii* | 2187 | ADC-58, OXA-69 | 2 | 1 | ≤0.06 | 0.25 | ≤0.06 | 0.5 | ≤0.06 |
| Ab209 | *A. baumannii* | - | - | 1 | 1 | ≤0.06 | 0.25 | ≤0.06 | 0.5 | ≤0.06 |
| Ab213 | *A. baumannii* | - | - | 2 | 1 | ≤0.06 | 0.12 | ≤0.06 | 0.5 | ≤0.06 |
| Ab218 | *A. baumannii* | - | - | 2 | 2 | ≤0.06 | 0.25 | ≤0.06 | 1 | ≤0.06 |
| Ab220 | *A. baumannii* | - | - | 1 | 0.5 | ≤0.06 | 0.25 | ≤0.06 | 0.5 | ≤0.06 |

Enmetazobactam was used at a fixed concentration of 8 mg/L. ETZ, enmetazobactam; FEP, cefepime; IMI, imipenem, SUL, sulbactam.

**Supplementary Table 3.** MIC (mg/L) values for the antibiotics tested against the collection of *A.* non-*baumannii* isolates (n = 87).

| **Isolate number** | **Species** | **β-lactamases** | **ETZ** | **FEP** | **FEP/ETZ** | **IMI** | **IMI/ETZ** | **SUL** | **SUL/ETZ** |
| --- | --- | --- | --- | --- | --- | --- | --- | --- | --- |
| NO-Ab2 | *Acinetobacter dispersus* | - | 2 | 0.5 | ≤0.06 | ≤0.06 | ≤0.06 | 0.5 | ≤0.06 |
| NO-Ab3 | *Acinetobacter ursingii* | - | 0.25 | 0.25 | ≤0.06 | ≤0.06 | ≤0.06 | 0.25 | ≤0.06 |
| NO-Ab27 | *Acinetobacter baylyi* | - | 2 | 0.5 | ≤0.06 | 0.12 | ≤0.06 | 1 | ≤0.06 |
| NO-Ab43 | *Acinetobacter nosocomialis* | - | 2 | 2 | ≤0.06 | ≤0.06 | ≤0.06 | 2 | ≤0.06 |
| NO-Ab59 | *Acinetobacter pittii* | - | 2 | 2 | ≤0.06 | 0.25 | ≤0.06 | 1 | ≤0.06 |
| NO-Ab60 | *Acinetobacter dijkshoorniae* | - | 4 | 2 | ≤0.06 | 0.25 | ≤0.06 | 1 | ≤0.06 |
| NO-Ab63 | *Acinetobacter dijkshoorniae* | - | 4 | 2 | ≤0.06 | 0.25 | ≤0.06 | 1 | ≤0.06 |
| NO-Ab64 | *Acinetobacter ursingii* | - | 1 | 1 | ≤0.06 | ≤0.06 | ≤0.06 | 1 | ≤0.06 |
| NO-Ab65 | *Acinetobacter dijkshoorniae* | - | 4 | 2 | ≤0.06 | 0.25 | ≤0.06 | 1 | ≤0.06 |
| NO-Ab66 | *Acinetobacter pittii* | - | 2 | 8 | ≤0.06 | 0.25 | ≤0.06 | 1 | ≤0.06 |
| NO-Ab68 | *Acinetobacter nosocomialis* | OXA-24/40 | 16 | 4 | 2 | 32 | 8 | 1 | 1 |
| NO-Ab69 | *Acinetobacter guillouiae* | - | 2 | 2 | ≤0.06 | ≤0.06 | ≤0.06 | 1 | ≤0.06 |
| NO-Ab70 | *Acinetobacter bereziniae* | - | 1 | 1 | ≤0.06 | 0.12 | ≤0.06 | 0.5 | ≤0.06 |
| NO-Ab73 | *Acinetobacter pittii* | - | 2 | 2 | ≤0.06 | ≤0.06 | ≤0.06 | 0.5 | ≤0.06 |
| NO-Ab74 | *Acinetobacter guillouiae* | - | 2 | 2 | ≤0.06 | 0.25 | ≤0.06 | 0.5 | ≤0.06 |
| NO-Ab75 | *Acinetobacter pittii* | - | 2 | 4 | ≤0.06 | 0.25 | ≤0.06 | 1 | ≤0.06 |
| NO-Ab76 | *Acinetobacter junii* | - | 2 | 0.5 | ≤0.06 | ≤0.06 | ≤0.06 | 0.5 | ≤0.06 |
| NO-Ab77 | *Acinetobacter junii* | OXA-24/40 | 2 | 1 | ≤0.06 | 32 | ≤0.06 | 0.5 | ≤0.06 |
| NO-Ab78 | *Acinetobacter pittii* | - | 2 | 1 | ≤0.06 | ≤0.06 | ≤0.06 | 0.5 | ≤0.06 |
| NO-Ab82 | *Acinetobacter pittii* | - | 2 | 1 | ≤0.06 | ≤0.06 | ≤0.06 | 0.5 | ≤0.06 |
| NO-Ab84 | *Acinetobacter bereziniae* | - | 1 | 1 | ≤0.06 | 0.12 | ≤0.06 | 1 | ≤0.06 |
| NO-Ab85 | *Acinetobacter calcoaceticus* | - | 4 | 4 | ≤0.06 | 0.12 | ≤0.06 | 2 | ≤0.06 |
| NO-Ab86 | *Acinetobacter dijkshoorniae* | - | 2 | 1 | ≤0.06 | 0.12 | ≤0.06 | 1 | ≤0.06 |
| NO-Ab87 | *Acinetobacter ursingii* | - | 0.5 | 0.25 | ≤0.06 | ≤0.06 | ≤0.06 | 0.12 | ≤0.06 |
| NO-Ab88 | *Acinetobacter dijkshoorniae* | - | 2 | 2 | ≤0.06 | 0.12 | ≤0.06 | 0.5 | ≤0.06 |
| NO-Ab89 | *Acinetobacter pittii* | - | 2 | 1 | ≤0.06 | 0.12 | ≤0.06 | 0.5 | ≤0.06 |
| NO-Ab90 | *Acinetobacter pittii* | - | 2 | 1 | ≤0.06 | ≤0.06 | ≤0.06 | 0.5 | ≤0.06 |
| NO-Ab91 | *Acinetobacter dijkshoorniae* | - | 2 | 2 | ≤0.06 | 0.12 | ≤0.06 | 1 | ≤0.06 |
| NO-Ab92 | *Acinetobacter haemolyticus* | - | 1 | 2 | ≤0.06 | 0.12 | ≤0.06 | 0.5 | ≤0.06 |
| NO-Ab94 | *Acinetobacter calcoaceticus* | - | 2 | 2 | ≤0.06 | ≤0.06 | ≤0.06 | 2 | ≤0.06 |
| NO-Ab97 | *Acinetobacter pittii* | - | 2 | 4 | ≤0.06 | ≤0.06 | ≤0.06 | 1 | ≤0.06 |
| NO-Ab98 | *Acinetobacter nosocomialis* | - | 2 | 2 | ≤0.06 | 0.12 | ≤0.06 | 2 | ≤0.06 |
| NO-Ab99 | *Acinetobacter proteolyticus* | - | 2 | 4 | ≤0.06 | 0.25 | ≤0.06 | 1 | ≤0.06 |
| NO-Ab101 | *Acinetobacter johnsonii* | - | 4 | 4 | ≤0.06 | 0.12 | ≤0.06 | 1 | ≤0.06 |
| NO-Ab102 | *Acinetobacter proteolyticus* | - | 4 | 4 | ≤0.06 | 0.25 | ≤0.06 | 1 | ≤0.06 |
| NO-Ab103 | *Acinetobacter ursingii* | - | 0.5 | 2 | ≤0.06 | ≤0.06 | ≤0.06 | 0.5 | ≤0.06 |
| NO-Ab104 | *Acinetobacter bereziniae* | - | 1 | 0.5 | ≤0.06 | 0.12 | ≤0.06 | 0.5 | ≤0.06 |
| NO-Ab105 | *Acinetobacter pittii* | - | 2 | 1 | ≤0.06 | 0.12 | ≤0.06 | 1 | ≤0.06 |
| NO-Ab106 | *Acinetobacter pittii* | - | 2 | 1 | ≤0.06 | ≤0.06 | ≤0.06 | 0.5 | ≤0.06 |
| NO-Ab107 | *Acinetobacter pittii* | - | 1 | 0.5 | ≤0.06 | ≤0.06 | ≤0.06 | 0.5 | ≤0.06 |
| NO-Ab108 | *Acinetobacter calcoaceticus* | - | 4 | 4 | ≤0.06 | ≤0.06 | ≤0.06 | 4 | ≤0.06 |
| NO-Ab111 | *Acinetobacter pittii* | - | 2 | 1 | ≤0.06 | 0.12 | ≤0.06 | 1 | ≤0.06 |
| NO-Ab113 | *Acinetobacter pittii* | - | 2 | 2 | ≤0.06 | 0.25 | ≤0.06 | 1 | ≤0.06 |
| NO-Ab114 | *Acinetobacter bereziniae* | - | 1 | 1 | ≤0.06 | 0.12 | ≤0.06 | 1 | ≤0.06 |
| NO-Ab115 | *Acinetobacter bereziniae* | - | 1 | 1 | ≤0.06 | 0.25 | ≤0.06 | 1 | ≤0.06 |
| NO-Ab116 | *Acinetobacter bereziniae* | - | 1 | 1 | ≤0.06 | 0.25 | ≤0.06 | 0.5 | ≤0.06 |
| NO-Ab117 | *Acinetobacter bereziniae* | - | 2 | 4 | ≤0.06 | 0.12 | ≤0.06 | 1 | ≤0.06 |
| NO-Ab118 | *Acinetobacter pittii* | - | 2 | 2 | ≤0.06 | 0.12 | ≤0.06 | 2 | ≤0.06 |
| NO-Ab119 | *Acinetobacter ursingii* | - | 8 | 8 | ≤0.06 | 0.25 | ≤0.06 | 0.5 | ≤0.06 |
| NO-Ab120 | *Acinetobacter ursingii* | - | 1 | 4 | ≤0.06 | ≤0.06 | ≤0.06 | 0.5 | ≤0.06 |
| NO-Ab121 | *Acinetobacter dijkshoorniae* | - | 2 | 4 | ≤0.06 | 0.12 | ≤0.06 | 2 | ≤0.06 |
| NO-Ab122 | *Acinetobacter beijerinckii* | - | 2 | 2 | ≤0.06 | 0.12 | ≤0.06 | 2 | ≤0.06 |
| NO-Ab123 | *Acinetobacter ursingii* | - | 2 | 2 | ≤0.06 | ≤0.06 | ≤0.06 | 2 | ≤0.06 |
| NO-Ab125 | *Acinetobacter dijkshoorniae* | - | 1 | 2 | ≤0.06 | ≤0.06 | ≤0.06 | 1 | ≤0.06 |
| NO-Ab126 | *Acinetobacter ursingii* | - | 0.5 | 4 | ≤0.06 | 0.12 | ≤0.06 | 1 | ≤0.06 |
| NO-Ab129 | *Acinetobacter bereziniae* | - | 1 | 1 | ≤0.06 | 0.25 | ≤0.06 | 1 | ≤0.06 |
| NO-Ab135 | *Acinetobacter pittii* | - | 2 | 1 | ≤0.06 | 0.12 | ≤0.06 | 0.5 | ≤0.06 |
| NO-Ab145 | *Acinetobacter pittii* | - | 2 | 2 | ≤0.06 | 0.12 | ≤0.06 | 1 | ≤0.06 |
| NO-Ab146 | *Acinetobacter iwoffii* | - | 4 | 2 | ≤0.06 | 0.25 | ≤0.06 | 2 | ≤0.06 |
| NO-Ab147 | *Acinetobacter ursingii* | - | 4 | 2 | ≤0.06 | 0.25 | ≤0.06 | 2 | ≤0.06 |
| NO-Ab151 | *Acinetobacter pittii* | - | 4 | 2 | ≤0.06 | 0.25 | ≤0.06 | 2 | ≤0.06 |
| NO-Ab154 | *Acinetobacter pittii* | - | 2 | 1 | ≤0.06 | 0.25 | ≤0.06 | 0.5 | ≤0.06 |
| NO-Ab155 | *Acinetobacter ursingii* | - | 4 | 4 | ≤0.06 | 0.25 | ≤0.06 | 1 | ≤0.06 |
| NO-Ab156 | *Acinetobacter bereziniae* | - | 4 | 1 | ≤0.06 | 0.12 | ≤0.06 | 1 | ≤0.06 |
| NO-Ab157 | *Acinetobacter pittii* | - | 4 | 4 | ≤0.06 | 0.25 | ≤0.06 | 1 | ≤0.06 |
| NO-Ab159 | *Acinetobacter iwoffii* | - | 2 | 0.5 | ≤0.06 | ≤0.06 | ≤0.06 | 0.5 | ≤0.06 |
| NO-Ab160 | *Acinetobacter dijkshoorniae* | - | 4 | 0.5 | ≤0.06 | 0.12 | ≤0.06 | 0.5 | ≤0.06 |
| NO-Ab161 | *Acinetobacter calcoaceticus* | - | 8 | 4 | ≤0.06 | 0.12 | ≤0.06 | 4 | ≤0.06 |
| NO-Ab162 | *Acinetobacter pittii* | - | 4 | 8 | ≤0.06 | 0.12 | ≤0.06 | 0.5 | ≤0.06 |
| NO-Ab164 | *Acinetobacter pittii* | - | 2 | 1 | ≤0.06 | ≤0.06 | ≤0.06 | 0.5 | ≤0.06 |
| NO-Ab165 | *Acinetobacter ursingii* | - | 2 | 2 | ≤0.06 | 0.12 | ≤0.06 | 1 | ≤0.06 |
| NO-Ab166 | *Acinetobacter ursingii* | - | 0.5 | 1 | ≤0.06 | ≤0.06 | ≤0.06 | 0.25 | ≤0.06 |
| NO-Ab175 | *Acinetobacter radioresistens* | - | 1 | 0.25 | ≤0.06 | 0.25 | ≤0.06 | 1 | ≤0.06 |
| NO-Ab177 | *Acinetobacter haemolyticus* | - | 1 | 1 | ≤0.06 | 0.12 | ≤0.06 | 0.5 | ≤0.06 |
| NO-Ab178 | *Acinetobacter pittii* | - | 2 | 4 | ≤0.06 | 0.12 | ≤0.06 | 0.5 | ≤0.06 |
| NO-Ab197 | *Acinetobacter pittii* | - | 2 | 2 | ≤0.06 | 0.12 | ≤0.06 | 0.5 | ≤0.06 |
| NO-Ab198 | *Acinetobacter calcoaceticus* | - | 4 | 4 | ≤0.06 | ≤0.06 | ≤0.06 | 2 | ≤0.06 |
| NO-Ab205 | *Acinetobacter calcoaceticus* | - | 4 | 4 | ≤0.06 | 0.25 | ≤0.06 | 2 | ≤0.06 |
| NO-Ab208 | *Acinetobacter nosocomialis* | - | 2 | 2 | ≤0.06 | 0.12 | ≤0.06 | 0.5 | ≤0.06 |
| NO-Ab210 | *Acinetobacter proteolyticus* | - | 1 | 1 | ≤0.06 | ≤0.06 | ≤0.06 | 0.25 | ≤0.06 |
| NO-Ab211 | *Acinetobacter calcoaceticus* | - | 4 | 2 | ≤0.06 | 0.12 | ≤0.06 | 1 | ≤0.06 |
| NO-Ab214 | *Acinetobacter ursingii* | - | 0.5 | 4 | ≤0.06 | ≤0.06 | ≤0.06 | ≤0.06 | ≤0.06 |
| NO-Ab215 | *Acinetobacter radioresistens* | - | 0.5 | 0.12 | ≤0.06 | 0.12 | ≤0.06 | 0.25 | ≤0.06 |
| NO-Ab216 | *Acinetobacter courvalinii* | - | 1 | 1 | ≤0.06 | 0.5 | ≤0.06 | 0.5 | ≤0.06 |
| NO-Ab217 | *Acinetobacter haemolyticus* | - | 0.5 | 2 | ≤0.06 | 0.12 | ≤0.06 | 0.25 | ≤0.06 |
| NO-Ab221 | *Acinetobacter ursingii* | - | 1 | 4 | ≤0.06 | ≤0.06 | ≤0.06 | 0.5 | ≤0.06 |
| NO-Ab222 | *Acinetobacter ursingii* | - | 1 | 1 | ≤0.06 | ≤0.06 | ≤0.06 | 0.5 | ≤0.06 |

Enmetazobactam was used at a fixed concentration of 8 mg/L. ETZ, enmetazobactam; FEP, cefepime; IMI, imipenem, SUL, sulbactam.

**Supplementary Table 4**. MIC and MBC (mg/L) values for enmetazobactam and sulbactam against a representative set of 9 clinical strains.

|  | **Isolate number** | **Species** | **ST** | **β-lactamases** | **ETZ** | | **SUL** | |
| --- | --- | --- | --- | --- | --- | --- | --- | --- |
|  |  |  |  |  | **MIC** | **MBC** | **MIC** | **MBC** |
| CHDLs-producing *A.* *baumannii* isolates | Ab4 | *A. baumannii* | 2 | ADC-30, OXA-66, IS*Aba1*-OXA-23 | 64 | 64 | 16 | 16 |
|  | Ab6 | *A. baumannii* | 2 | ADC-30, OXA-66, IS*Aba1*-OXA-23, TEM-12 | 64 | 64 | 16 | 16 |
|  | Ab9 | *A. baumannii* | 2 | ADC-30, OXA-66, OXA-23, TEM-12 | 8 | 8 | 4 | 4 |
| non-CHDLs-producing *A.* *baumannii* isolates | Ab1 | *A. baumannii* | 164 | ADC-6, OXA-91 | 2 | 4 | 0.5 | 1 |
|  | Ab11 | *A. baumannii* | 239 | ADC-2, OXA-51 | 2 | 16 | 1 | 2 |
|  | Ab34 | *A. baumannii* | 2182 | ADC-2, OXA-130 | 4 | 8 | 0.5 | 0.5 |
| *A.* non-*baumannii* isolates | NO-Ab84 | *A. bereziniae* | - | - | 1 | 2 | 1 | 1 |
|  | NO-Ab88 | *A. dijkshoorniae* | - | - | 2 | 2 | 0.5 | 0.5 |
|  | NO-Ab89 | *A. pittii* | - | - | 2 | 2 | 0.5 | 0.5 |

ETZ, enmetazobactam; SUL, sulbactam.

**Supplementary Table 5.** Synergy assays. i) MICs (mg/L) values for sulbactam and enmetazobactam alone and for the sulbactam/enmetazobactam combination. ii) MICs (mg/L) values for colistin and enmetazobactam alone and for the colistin/enmetazobactam combination. iii) MICs (mg/L) values for sulbactam and cefepime alone and in combination (both in presence of 8 mg/L of enmetazobactam).

| **Isolate** | **CHDL** | **SUL** | ^a^**SUL _ETZ_** | **ETZ** | **ETZ _SUL_** | **FIC_index_** | **Synergy (Y/N)** |
| --- | --- | --- | --- | --- | --- | --- | --- |
| **Ab4** | OXA-23 | 16 | 16 | 128 | 64 | 1.5 | No |
| **Ab138** | OXA-58 | 8 | 4 | 32 | 16 | 1 | No |
| **Ab180** | OXA-24/40 | 2 | 1 | 16 | 4 | 0.75 | No |
|  |  |  |  |  |  |  |  |
| **Isolate** | **CHDL** | **COL** | ^b^**COL _ETZ_** | **ETZ** | **ETZ _COL_** | **FIC_index_** | **Synergy (Y/N)** |
| **Ab4** | OXA-23 | 1 | 0,5 | 64 | 16 | 0.75 | No |
| **Ab138** | OXA-58 | 1 | 0.5 | 32 | 16 | 1 | No |
| **Ab180** | OXA-24/40 | 0.25 | 0.12 | 16 | 1 | 0.55 | No |
|  |  |  |  |  |  |  |  |
| **Isolate** | **CHDL** | **SUL _(ETZ)_** | ^c^**SUL_FEP (ETZ)_** | **FEP _(ETZ)_** | **FEP_SUL (ETZ)_** | **FIC_index_** | **Synergy (Y/N)** |
| **Ab4** | OXA-23 | 16 | 8 | 32 | 16 | 1 | No |
| **Ab138** | OXA-58 | 4 | 2 | 16 | 4 | 0.75 | No |
| **Ab180** | OXA-24/40 | 1 | 0.5 | 8 | 8 | 1.5 | No |

^a^ SUL_ETZ_, sulbactam MICs in the presence of enmetazobactam; ETZ_SUL_, enmetazobactam MICs in the presence of sulbactam. ^b^ COL_ETZ_, colistin MICs in the presence of enmetazobactam; ETZ_COL_, enmetazobactam MICs in the presence of colistin. ^c^ SUL_FEP (ETZ 8 mg/L)_, sulbactam MICs in the presence of cefepime; FEP_SUL (ETZ 8 mg/L)_, cefepime MICs in the presence of sulbactam; synergy assays among sulbactam and cefepime were always performed in presence of enmetazobactam at a fixed concentration of 8 mg/L.

**Supplementary Table 6.** MIC (mg/L) values for enmetazobactam and sulbactam alone and combined with durlobactam against the collection of carbapenem-resistant *A. baumannii* isolates (n = 67).

| **Isolate number** | **Species** | **ST** | **β-lactamases** | **ETZ** | **ETZ/DUR** | **SUL** | **SUL/DUR** |
| --- | --- | --- | --- | --- | --- | --- | --- |
| Ab4 | *A. baumannii* | 2 | ADC-30, OXA-66, OXA-23 | 64 | 2 | 16 | 2 |
| Ab5 | *A. baumannii* | 2 | ADC-30, OXA-66, OXA-23 | 64 | 2 | 16 | 1 |
| Ab6 | *A. baumannii* | 2 | ADC-30, OXA-66, OXA-23, TEM-12 | 64 | 2 | 16 | 1 |
| Ab7 | *A. baumannii* | 2 | ADC-30, OXA-66, OXA-23, | 64 | 2 | 32 | 2 |
| Ab8 | *A. baumannii* | 2 | ADC-30, OXA-66, OXA-23, | 64 | 4 | 16 | 1 |
| Ab9 | *A. baumannii* | 2 | ADC-30, OXA-66, OXA-23, TEM-12 | 32 | 2 | 32 | 2 |
| Ab10 | *A. baumannii* | 2 | ADC-30, OXA-66, OXA-23 | 64 | 2 | 16 | 1 |
| Ab12 | *A. baumannii* | 2 | ADC-30, IS*Aba1*-OXA-201 | 4 | 2 | 4 | 1 |
| Ab13 | *A. baumannii* | 2 | ADC-30, IS*Aba1*-OXA-201 | 4 | 2 | 2 | 1 |
| Ab14 | *A. baumannii* | 2 | ADC-30, IS*Aba1*-OXA-201 | 4 | 2 | 2 | 1 |
| Ab15 | *A. baumannii* | 2 | ADC-30, IS*Aba1*-OXA-201 | 8 | 2 | 2 | 1 |
| Ab16 | *A. baumannii* | 2 | ADC-30, IS*Aba1*-OXA-201 | 8 | 2 | 2 | 1 |
| Ab17 | *A. baumannii* | 2 | ADC-30, IS*Aba1*-OXA-201 | 8 | 1 | 2 | 1 |
| Ab18 | *A. baumannii* | 2 | ADC-30, IS*Aba1*-OXA-201 | 4 | 2 | 2 | 1 |
| Ab19 | *A. baumannii* | 2 | ADC-30, OXA-66, OXA-58 | 8 | 2 | 8 | 0.25 |
| Ab20 | *A. baumannii* | 2 | ADC-30, IS*Aba1*-OXA-201 | 4 | 2 | 2 | 0.25 |
| Ab24 | *A. baumannii* | 2 | ADC-30, IS*Aba1*-OXA-201 | 4 | 4 | 2 | 1 |
| Ab25 | *A. baumannii* | 2 | ADC-30, IS*Aba1*-OXA-201 | 4 | 2 | 2 | 1 |
| Ab26 | *A. baumannii* | 2 | ADC-30, OXA-66, OXA-58 | 8 | 2 | 4 | 0.12 |
| Ab28 | *A. baumannii* | 2 | IS*Aba1*-ADC-30, OXA-66, OXA-23 | 64 | 2 | 16 | 1 |
| Ab29 | *A. baumannii* | 2 | ADC-30, OXA-66, OXA-23 | 64 | 2 | 32 | 1 |
| Ab30 | *A. baumannii* | 2 | IS*Aba1*-ADC-30, OXA-66, OXA-23 | 64 | 2 | 16 | 2 |
| Ab31 | *A. baumannii* | 2 | ADC-30, OXA-66, IS*Aba1*-OXA-23 | 64 | 2 | 16 | 1 |
| Ab32 | *A. baumannii* | 2 | IS*Aba1*-ADC-30, OXA-66, OXA-23 | 64 | 2 | 16 | 1 |
| Ab33 | *A. baumannii* | 2 | ADC-30, OXA-66, OXA-23 | 64 | 2 | 16 | 1 |
| Ab35 | *A. baumannii* | 2 | ADC-30, OXA-66, OXA-23 | 64 | 2 | 16 | 1 |
| Ab36 | *A. baumannii* | 2 | ADC-30, OXA-66, OXA-23 | 64 | 2 | 16 | 1 |
| Ab37 | *A. baumannii* | 2 | ADC-30, OXA-66, OXA-23 | 64 | 2 | 16 | 1 |
| Ab39 | *A. baumannii* | 2 | ADC-30, OXA-66, OXA-23 | 16 | 2 | 4 | 0.12 |
| Ab41 | *A. baumannii* | 2 | ADC-30, OXA-66, OXA-23 | 64 | 2 | 16 | 1 |
| Ab42 | *A. baumannii* | 2 | ADC-30, OXA-66, OXA-23 | 64 | 2 | 16 | 2 |
| Ab44 | *A. baumannii* | 2 | ADC-30, OXA-66, OXA-23 | 32 | 4 | 8 | 1 |
| Ab45 | *A. baumannii* | 1 | ADC-75, OXA-69, OXA-23 | 64 | 1 | 16 | 1 |
| Ab46 | *A. baumannii* | 1 | ADC-75, OXA-69, OXA-23 | 64 | 2 | 16 | 0.5 |
| Ab47 | *A. baumannii* | 1 | ADC-75, OXA-69, OXA-23 | 64 | 2 | 16 | 0.5 |
| Ab49 | *A. baumannii* | 1 | ADC-75, OXA-69, OXA-23 | 64 | 2 | 16 | 1 |
| Ab52 | *A. baumannii* | 1 | ADC-75, OXA-69, OXA-23 | 64 | 2 | 8 | 0.5 |
| Ab53 | *A. baumannii* | 1 | IS*Aba1*-ADC-75, OXA-69, OXA-23 | 64 | 2 | 16 | 0.5 |
| Ab54 | *A. baumannii* | 1 | ADC-75, OXA-69, OXA-23 | 64 | 2 | 16 | 0.5 |
| Ab55 | *A. baumannii* | 1 | ADC-75, OXA-69, OXA-23 | 64 | 2 | 8 | 0.5 |
| Ab56 | *A. baumannii* | 1 | ADC-75, OXA-69, OXA-23 | 64 | 0.5 | 8 | 0.25 |
| Ab83 | *A. baumannii* | 25 | ADC-5, OXA-64, OXA-23 | 64 | 2 | 32 | 0.5 |
| Ab109 | *A. baumannii* | 1 | ADC-75, OXA-69, OXA-23 | 32 | 2 | 16 | 1 |
| Ab133 | *A. baumannii* | 2 | ADC-30, OXA-66, OXA-58, TEM-12 | 8 | 2 | 32 | 0.5 |
| Ab134 | *A. baumannii* | 2 | ADC-30, OXA-66, OXA-58, TEM-12 | 8 | 2 | 32 | ≤0.06 |
| Ab137 | *A. baumannii* | 85 | ADC-2, OXA-94, OXA-23 | 64 | 2 | 16 | 1 |
| Ab138 | *A. baumannii* | 745 | ADC-30, OXA-66, OXA-58 | 64 | 4 | 4 | 0.5 |
| Ab140 | *A. baumannii* | 85 | ADC-2, OXA-94, OXA-23 | 64 | 4 | 16 | 2 |
| Ab141 | *A. baumannii* | 745 | ADC-30, OXA-66, OXA-58 | 8 | 1 | 8 | 0.5 |
| Ab144 | *A. baumannii* | 745 | ADC-30, OXA-66, OXA-58 | 32 | 2 | 8 | ≤0.06 |
| Ab169 | *A. baumannii* | 2 | ADC-30, OXA-66, OXA-23 | 32 | 2 | 16 | 1 |
| Ab171 | *A. baumannii* | 2 | ADC-30, OXA-66, OXA-23 | 32 | 2 | 16 | 0.5 |
| Ab173 | *A. baumannii* | 2 | IS*Aba1*-ADC-30, OXA-66, OXA-23 | 64 | 2 | 32 | 0.5 |
| Ab174 | *A. baumannii* | 2 | ADC-30, OXA-66, OXA-23 | 64 | 2 | 16 | 1 |
| Ab179 | *A. baumannii* | 2 | ADC-30, OXA-66, OXA-23 | 64 | 2 | 16 | 1 |
| Ab180 | *A. baumannii* | 2 | ADC-30, OXA-66, OXA-24/40 | 32 | 2 | 4 | ≤0.06 |
| Ab181 | *A. baumannii* | 2 | ADC-30, OXA-66, OXA-23 | 32 | 2 | 16 | 1 |
| Ab182 | *A. baumannii* | 2 | ADC-30, OXA-66, OXA-23 | 16 | 2 | 8 | 0.25 |
| Ab183 | *A. baumannii* | 2 | ADC-30, OXA-66, OXA-23 | 32 | 2 | 8 | 0.25 |
| Ab184 | *A. baumannii* | 2 | ADC-30, OXA-66, OXA-23 | 32 | 2 | 16 | 1 |
| Ab186 | *A. baumannii* | 2 | ADC-30, OXA-66, OXA-23 | 32 | 2 | 16 | 1 |
| Ab191 | *A. baumannii* | 2 | ADC-30, OXA-66, OXA-23 | 64 | 2 | 16 | 0.25 |
| Ab192 | *A. baumannii* | 2 | ADC-30, OXA-66, OXA-23 | 32 | 2 | 16 | 0.5 |
| Ab194 | *A. baumannii* | 2 | ADC-30, OXA-66, OXA-23 | 64 | 2 | 16 | 1 |
| Ab199 | *A. baumannii* | 2 | ADC-30, OXA-66, OXA-23 | 32 | 2 | 16 | 0.5 |
| Ab201 | *A. baumannii* | 2 | ADC-30, OXA-66, OXA-23 | 64 | 4 | 16 | 1 |
| Ab204 | *A. baumannii* | 2 | ADC-30, OXA-66, OXA-23 | 32 | ≤0.06 | 16 | ≤0.06 |

Durlobactam was used at a fixed concentration of 4 mg/L. ETZ, enmetazobactam; SUL, sulbactam; DUR, durlobactam.

**Supplementary Table 7.** IC_50_ values of β-lactamase inhibitors against OXA-23.

| **Inhibitor** | **IC_50_ (µM)** |
| --- | --- |
| Enmetazobactam | 32.41 ± 1.85 |
| Sulbactam | 29.21 ± 4.10 |
| Durlobactam | 0.17 ± 0.034 |

**
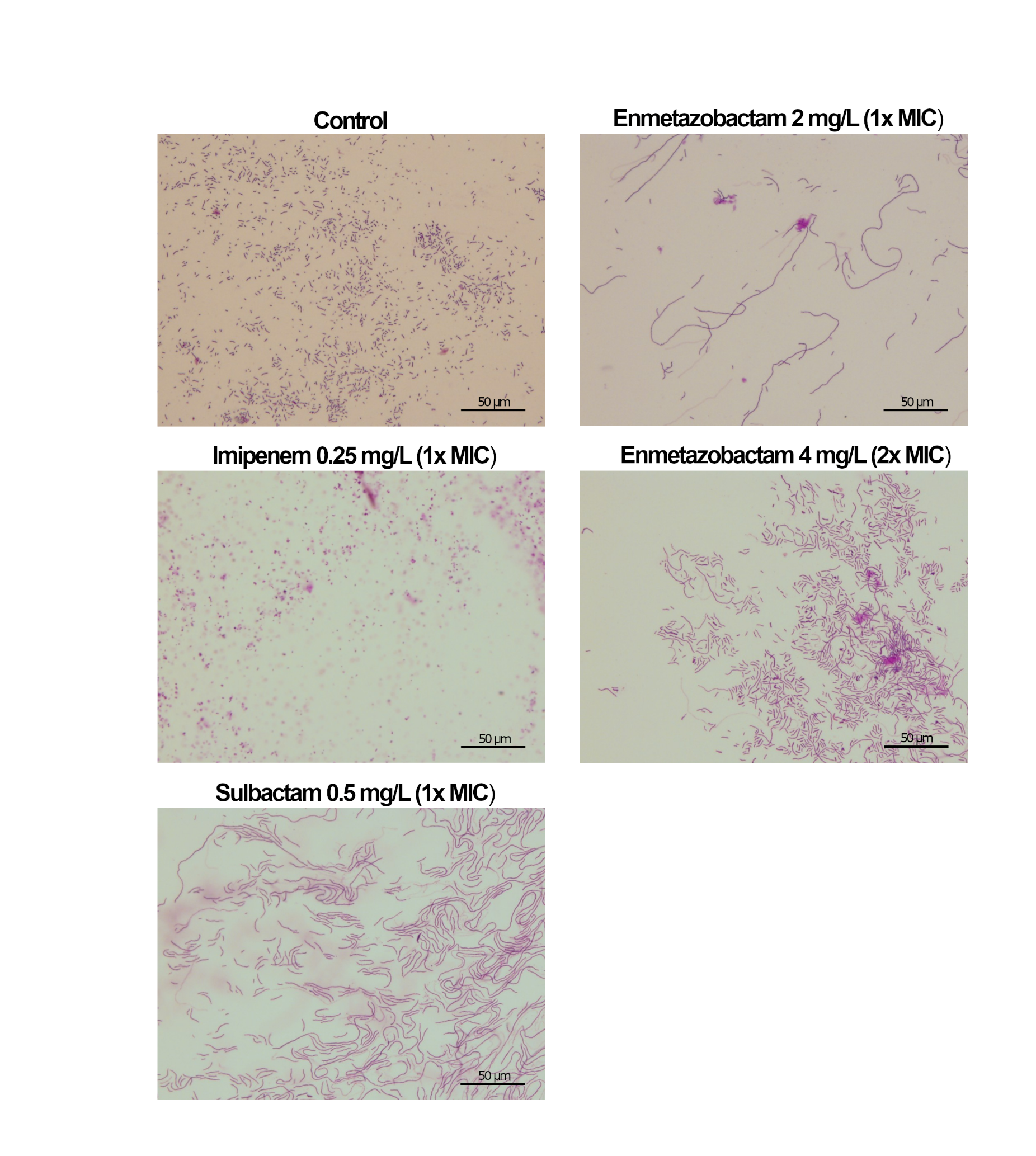
SUPPLEMENTARY FIGURES**

**Supplementary Figure 1.** Examples of cell morphology assays showing the appearance of untreated (control) and treated (with imipenem, sulbactam and enmetazobactam) *A. baumannii* ATCC 19606 cells.

**Supplementary Figure 2.** Dissociation experiments (*k*_off_) of OXA-23 with enmetazobactam, durlobactam and OXA-23 without β-lactamase inhibitor. The β-lactamase activity was determined by monitoring the increase in absorbance in the UV-Vis spectrum due to nitrocefin hydrolysis.


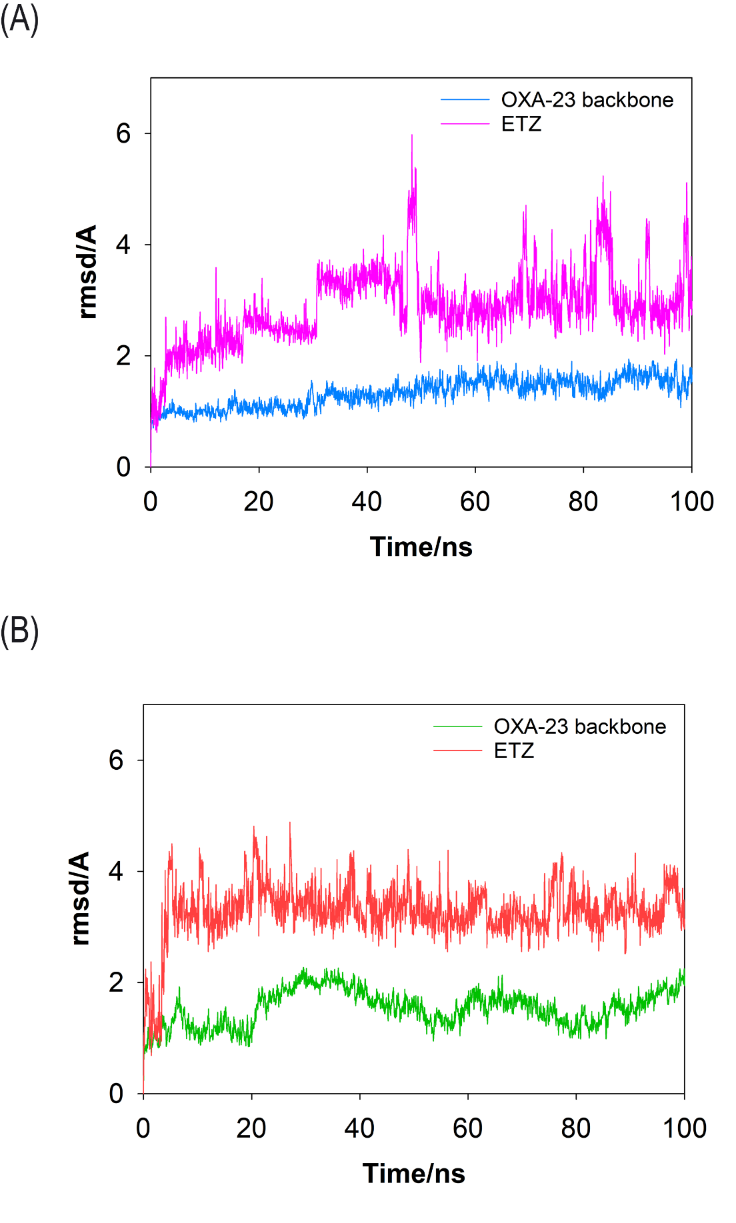


**Supplementary Figure 3.** Root-mean-square deviation (rmsd) plots for the enzyme backbone (Cα, C, O and N atoms) and enmetazobactam (ETZ), calculated from the MD simulations of the ETZ/OXA-23 Michaelis complex. Two complexes were constructed using the enzyme coordinates from PDB entries 4JF4 (A) and 9NSW (B), yielding similar results. Notably, the ligand remains stably positioned within the active site throughout the entire simulation.


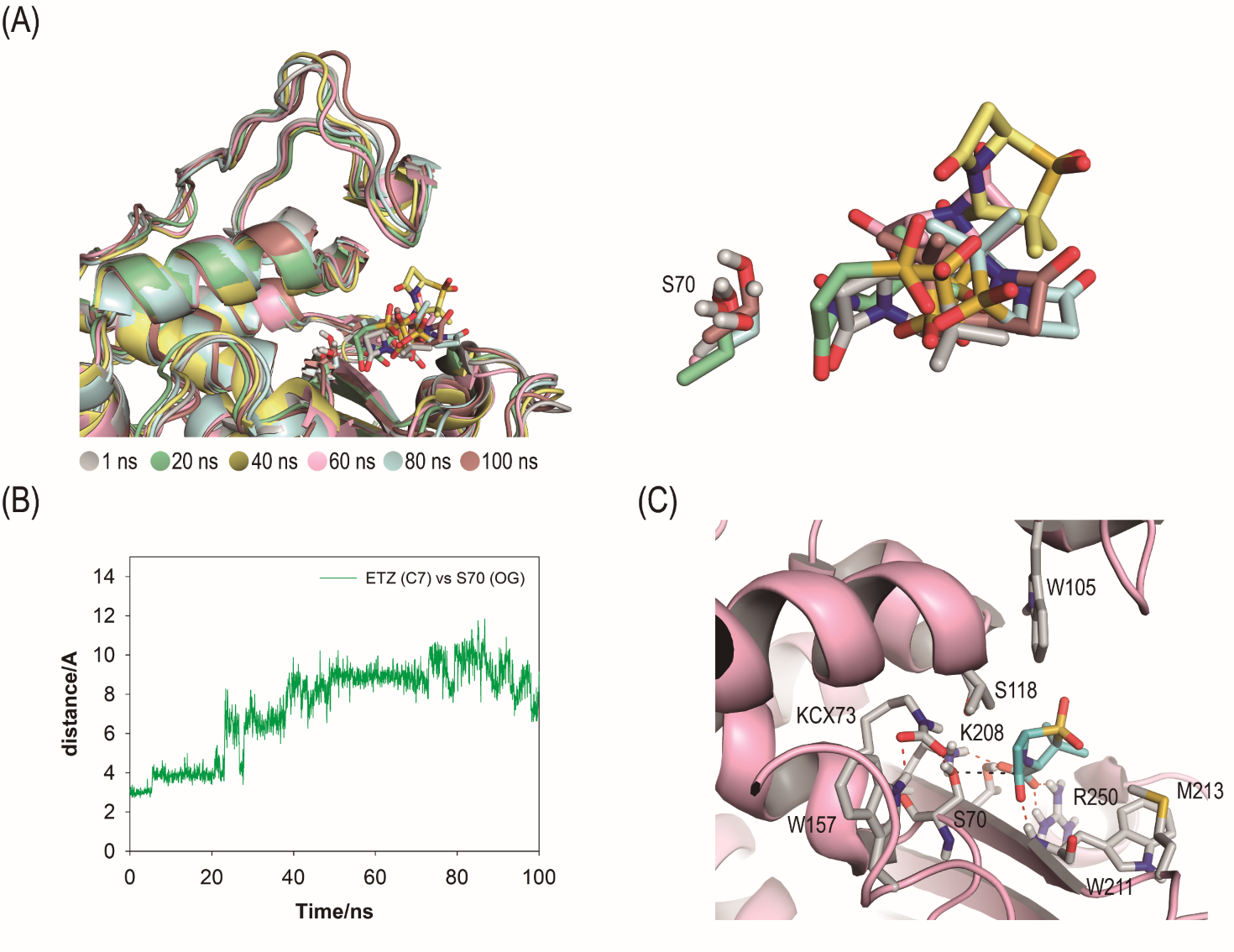

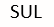


**Supplementary Figure 4.** (A) Comparison of several snapshots of the sulbactam/OXA-23 Michaelis complex during 100 ns of dynamic simulation. Close view of sulbactam arrangement relative to the catalytic serine residue (S70) is also provided. Note the high mobility of the ligand during the simulation. (B) Variation of the relative distance between the carbonyl group (C7 atom) in the ligand and S70 (OG atom) during the entire simulation. A noticeable displacement of the ligand from the catalytic serine residue is observed, preventing effective covalent bond formation. (C) Sulbactam binding mode in the OXA-23 active site at the early stage of the simulation (20 ns), showing a conformation suitable for the formation of the acyl-enzyme adduct.

**
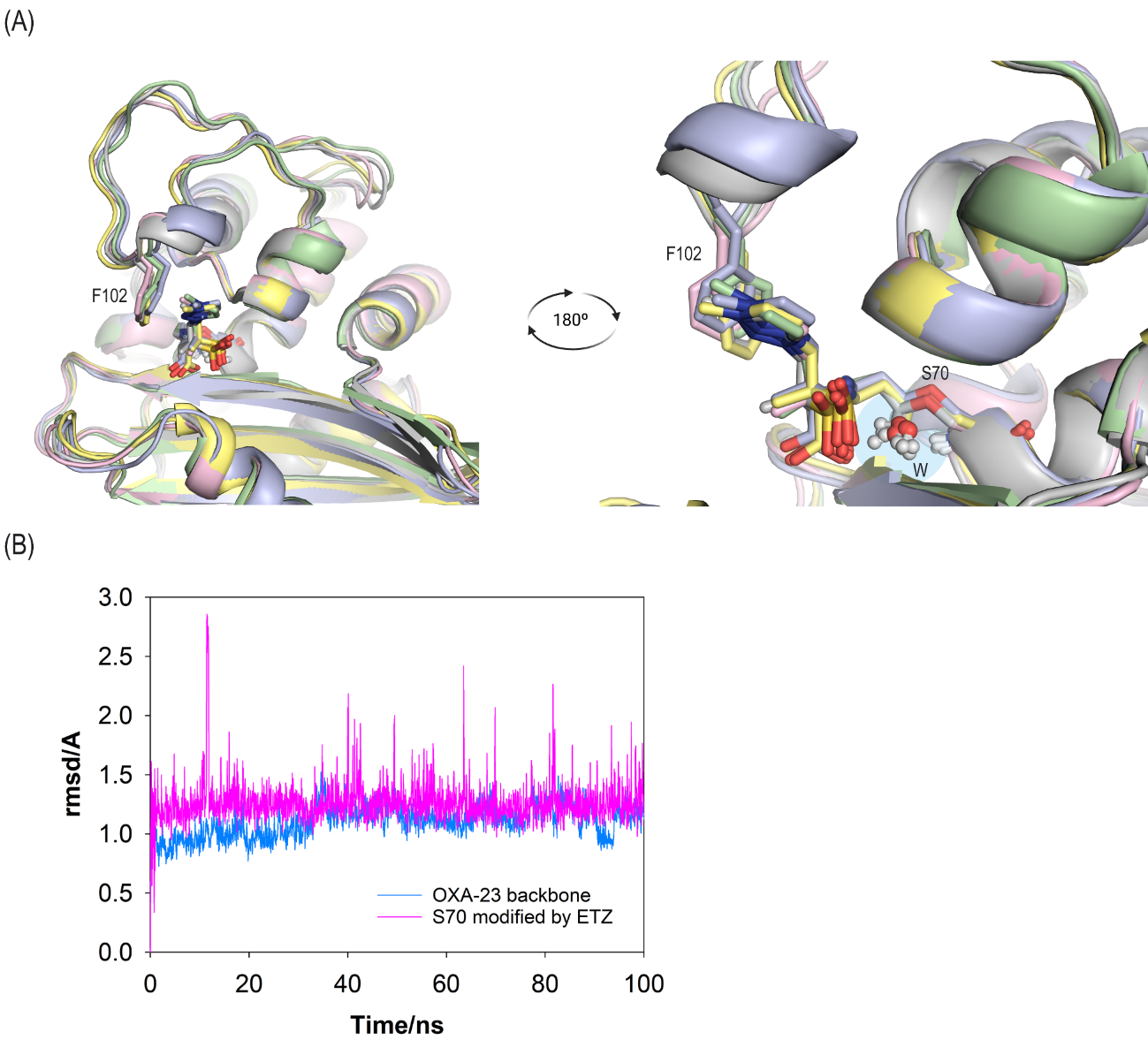
**

**Supplementary Figure 5.** (A) Comparison of several snapshots of the enmetazobactam/OXA-23 acyl-enzyme adduct during 100 ns of dynamic simulation. Two views are shown. Note how the triazole moiety in the modified substrate would establish a strong anion-π interaction with the essential residue F102, which is involved in the enzyme tunnel-like entrance. This arrangement would also facilitate entrapment of a water molecule in an arrangement suitable for nucleophilic attack to the carboxylate group in the modified catalytic serine residue, which remained stable throughout the whole simulation. (B) Root-mean-square deviation (rmsd) plots for the enzyme backbone (Cα, C, O and N atoms) and modified catalytic serine residue calculated from the MD simulations of enzyme adduct. The low average rmsd values (1.1 Å for the enzyme backbone and 1.3 Å for the modified enmetazobactam), together with the irrelevant differences in the whole structure, reveal the high stability of the conformation of the resulting adduct.

**
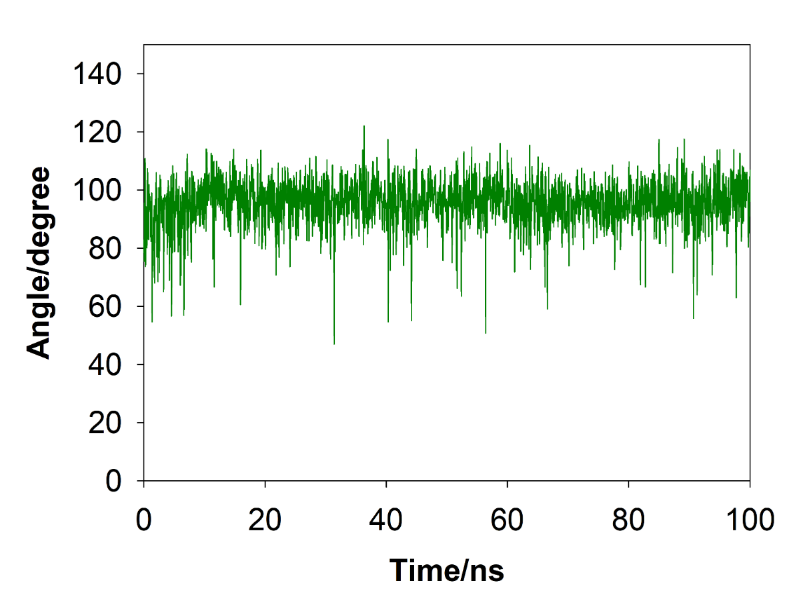
**

**Supplementary Figure 6.** Variation in the angle of attack of the oxygen atom in the hydrolytic water (W1) to the carboxylate group (C7 atom) in the acyl-enzyme adduct (residue S70) throughout the whole simulation. An average degree value of 95º was obtained.

**
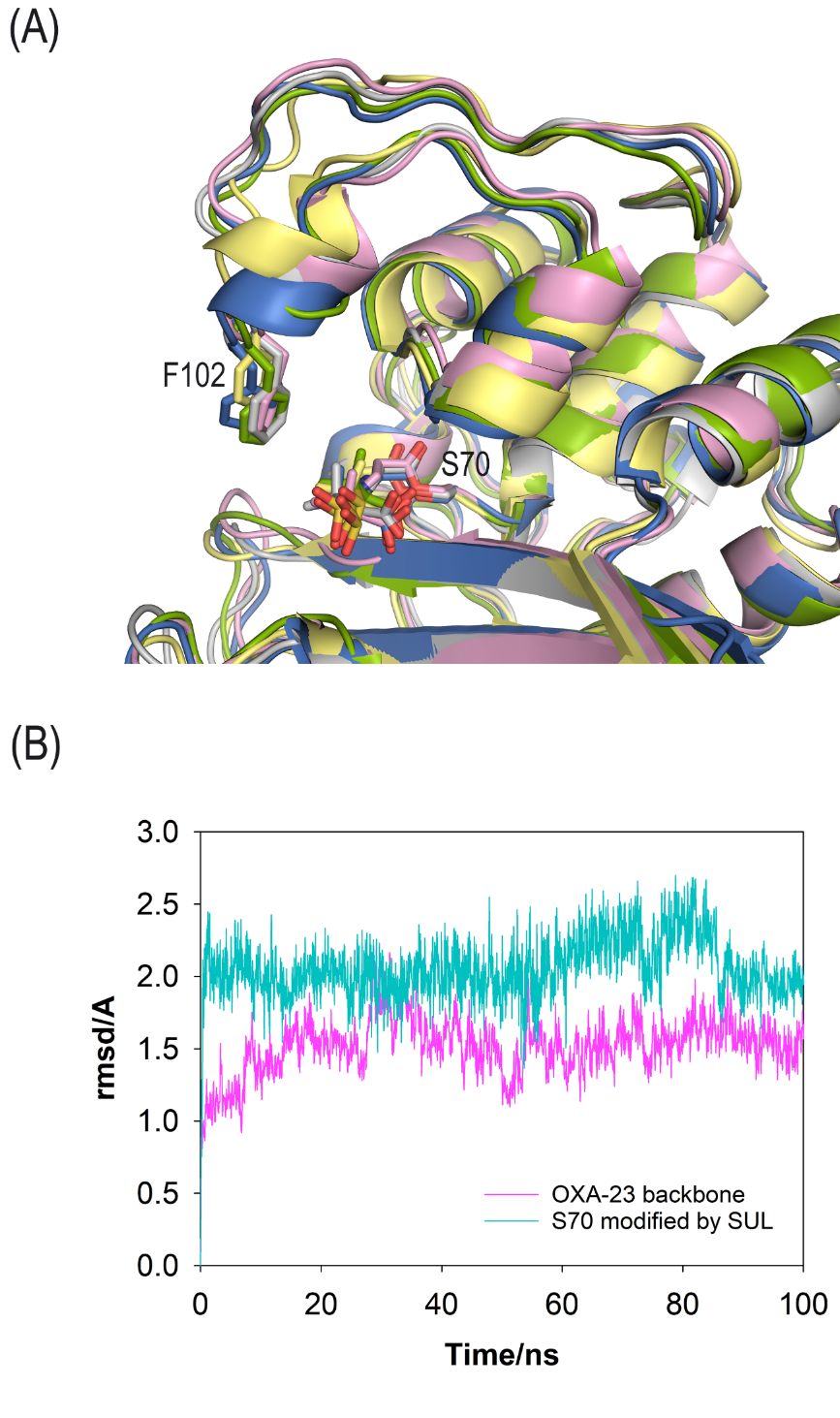
**

**Supplementary Figure 7.** (A) Comparison of several snapshots of the sulbactam/OXA-23 enzyme adduct during 100 ns of dynamic simulation. Note how relevant motion of the acyl-serine residue was observed and no significant interactions with residue F102 were detected. (B) Root-mean-square deviation (rmsd) plots for the OXA-23 enzyme backbone (Cα, C, O and N atoms) and the catalytic serine residue S70 covalently modified by sulbactam calculated from the MD simulations of the enzyme adduct. Average rmsd values of 2.1 Å (acyl-serine residue) and 1.5 Å (enzyme backbone) were obtained, which are larger than those obtained for the enmetazobactam/OXA-23 enzyme adduct (see Supplementary Figure 5).
